# Electromechanical modelling and simulation of human‐induced pluripotent stem cell‐derived cardiomyocytes predict drug‐induced contractility effects

**DOI:** 10.1113/JP288623

**Published:** 2026-03-11

**Authors:** Milda Folkmanaite, Xin Zhou, Andreas Koschinski, Wan‐Hua Hong, Marilù Casini, Manuel Koch, Ursula Ravens, Remi Peyronnet, Manuela Zaccolo, Blanca Rodriguez

**Affiliations:** ^1^ University of Oxford Oxford United Kingdom; ^2^ University of Turin Turin Italy; ^3^ Institute for Experimental Cardiovascular Medicine University Heart Center Freiburg – Bad Krozingen, Medical Center – University of Freiburg and Faculty of Medicine, University of Freiburg Freiburg Germany

**Keywords:** cardiomyocyte, cardiovascular physiology, computer modelling, stem cell

## Abstract

**Abstract:**

Human‐induced pluripotent stem cell‐derived cardiomyocytes (hiPSC‐CMs) hold promise in personalized medicine, particularly for cardiac diseases and human‐data‐based pharmacology studies. Assessing hiPSC‐CM mechanics and their changes in response to drug action *in silico* enables more efficient drug testing. For such investigations, hiPSC‐CMs also provide a versatile alternative to adult human cardiac tissue which is limited in availability for research. To enable *in silico* investigations of hiPSC‐CM electrophysiology and contraction, we developed and evaluated two versions of hiPSC‐CM electromechanical models with different maturation states. The models were based solely on human cardiomyocyte and hiPSC‐CM data. The evaluation process involved comparing simulation outcomes with an extensive dataset of experimental data to ensure the reliability of the model within the context of hiPSC‐CM pharmacology studies. The models uniquely incorporated the mechanical properties of hiPSC‐CMs, providing insights into the mechanisms underlying their contractile behaviour. In our *in silico* studies, we simulated the effects of 64 different drugs, including those with previously untested inotropic effects. We demonstrated agreement between the simulation and experimental datasets, correctly identifying the inotropic effects of 41 out of 48 drugs. We also compared the effect of pharmacological agents with unknown inotropic effects and conducted novel experiments demonstrating agreement with simulation outcomes. Finally, using the models, we demonstrated the mechanisms of previously unrecognized rate‐dependent inotropic effects of paliperidone. Altogether this study presents an *in vitro* – *in silico* framework which is evaluated against experimental data and allows for simulating drug‐dependent electromechanical effects with high accuracy and prediction of rate‐dependent inotropic effects.

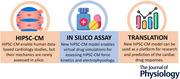

**Key points:**

- Human‐induced pluripotent stem cell‐derived cardiomyocytes (hiPSC‐CMs) are promising for drug testing and disease modelling, but current computer models that allow us to simulate hiPSC‐CM behaviour lack human‐specific mechanical properties.
- We developed and validated hiPSC‐CM electromechanical models, allowing accurate simulations of contraction, calcium signalling and electrophysiology for two different maturation stages.
- Simulations with the new models correctly predicted inotropic effects for 41 out of 48 drugs and identified previously unknown effects of two drugs, later confirmed experimentally.
- Simulations revealed novel rate‐dependent inotropic effects of paliperidone linked to calcium handling differences in paced *versus* non‐paced cells.
- This *in silico* framework can enhance drug testing accuracy and understanding through mechanistic studies by integrating experimental data with computational predictions.

## Graphical abstract

**Abstract figure legend** A study described in this paper describes a new tool for hiPSC‐CM electromechanical behaviour simulations using mathematical modelling. The model enables *in silico* assays making drug testing quick, comprehensive and accurate. New hiPSC‐CM models can be used as a platform to integrate *in vitro* and *in silico* findings.

## Introduction

Human‐induced pluripotent stem cell‐derived cardiomyocytes (hiPSC‐CMs) have emerged as crucial tools in cardiovascular research, providing a patient‐specific platform for drug testing and disease modelling (Li et al., 2020; Zhu et al., 2023). hiPSC‐CMs offer several advantages over adult human cardiomyocytes, including the possibility to generate large quantities of cells and the potential for genetic manipulation, which is particularly beneficial for studying genetic diseases. Patient‐derived hiPSC‐CMs allow the study of disease mechanisms in a personalized context. This patient‐specificity enables the development of precision therapies tailored to individual genetic profiles. Unlike adult cardiomyocytes, which are challenging to obtain and maintain in culture, hiPSC‐CMs provide a robust and renewable cell source that can be propagated and used in high‐throughput screening assays, facilitating drug discovery and toxicology studies. However, hiPSC‐CMs also have limitations, such as immature phenotypes compared to adult cardiomyocytes.

The Comprehensive in Vitro Proarrhythmia Assay (CiPA) initiative highlights the importance of assessing drug‐induced effects using the combination of *in silico* approaches and wet‐lab‐based assays (Colatsky et al., 2016). While many computational studies traditionally focus on the electrical properties of these cells (Koivumäki et al., 2018; Kernik et al., 2019; Paci et al., 2020, 2021), there is a significant gap in understanding their mechanical responses, such as contraction and relaxation, which are essential for evaluating heart medications and elucidating mechanisms of heart disease. Developing models that include contractility is crucial because mechanical properties play a vital role in assessing the efficacy and safety of cardiac drugs. Although contractility can be measured experimentally, *in silico* models allow for more efficient, scalable and cost‐effective drug testing, and they enable the exploration of scenarios that might be challenging to replicate experimentally, for example, due to limited tissue availability (Margara et al., 2021; Bartolucci et al., 2022). However, existing electromechanical models (Forouzandehmehr et al., 2021) build on previously developed contractile formulations and adapt them through reparameterisation to match hiPSC‐CM experimental data, rather than employing contractile model structures specifically developed to represent human or hiPSC‐CM contractile physiology. [Correction made on 22 May 2026, after first online publication: the preceding sentence has been updated to clarify that the electromechanical hiPSC‐CM model of Forouzandehmehr et al. (2021) relies on an established contractile formulation that is adapted using hiPSC‐derived measurements.]

To address this gap, we have developed hiPSC‐CM electromechanical models corresponding to two different maturation stages to simulate both electrophysiological and contractile behaviours of hiPSC‐CMs under various pharmacological conditions. The credibility of the model is based on the human data and knowledge integrated in the equations and parameters, and also the comparison of simulation results against experimental data, including: hiPSC‐CM contractility (contraction and relaxation duration, peak amplitude), calcium signalling (calcium transient duration), action potentials (action potential duration) (Schwan et al., 2016; Sewanan et al., 2019; Forouzandehmehr et al., 2021) and drug effects observed experimentally for 64 different compounds, including those with both known and unknown effects on hiPSC‐CM behaviour. Finally, for drugs previously untested in hiPSC‐CMs, our model can be used to successfully predict inotropic effects – how drugs increase or decrease the force of contraction – which are then later confirmed using hiPSC‐CMs through targeted experimental validations in the wet‐lab. By providing a tool that integrates and extends sparse experimental data, the model provides a way to enhance the confidence in drug testing outcomes, particularly valuable in early‐stage drug development. Furthermore, our model serves as a platform for mechanistic studies, offering insights into the underlying molecular drivers of drug responses, which are crucial for designing safer and more effective cardiac therapies.

## Methods

### Construction of the hiPSC‐CMs electromechanical model

The previously published hiPSC‐CM electrophysiology Paci model was coupled with the human adult cell tension Land model (Land et al., 2017; Paci et al., 2020). The hiPSC‐CM electrophysiology Paci model includes formulations to describe different currents including fast Na^+^ current (*I*_Na_), the late Na^+^ current (*I*_NaL_), the funny current (*I*_f_), the L‐type Ca^2+^ current (*I*_CaL_), the transient outward K^+^ current (*I*_to_), the rapid and slow delayed rectifier K^+^ currents (*I*_Kr_ and *I*_Ks_), the inward rectifier K^+^ current (*I*_K1_), the Na^+^/Ca^2+^ exchanger (*I*_NCX_), the Na^+^/K^+^ pump (*I*_NaK_), the sarcolemmal Ca^2+^ pump (*I*_pCa_), and the Na^+^ and Ca^2+^ background currents (*I*_bNa_ and *I*_bCa_). The SR compartment exchanges Ca^2+^ with the cytosol through three fluxes: the RyR‐sensitive release current (*I*_rel_), the sarco‐endoplasmic reticulum calcium ATPase (SERCA) pump (*I*_up_) and the leakage current (*I*_leak_). It features two compartments, cytosol and sarcoplasmic reticulum, without t‐tubules as observed in hiPSC‐CMs. The model has been validated for drug action using experimental hiPSC‐CM data including calcium and action potential recordings (Paci et al., 2020), consistent with our present study.

**Figure 1:**
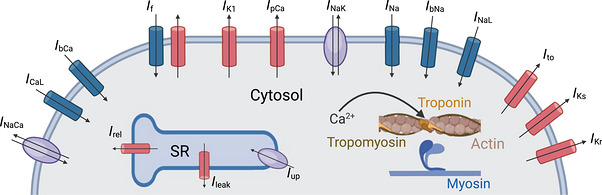
Schematic representation of the model Created with BioRender.

The Land model was used for active tension generation. It is based on measurements obtained from human cardiomyocytes at body temperature and features troponin C and tropomyosin kinetics as well as a three‐state cross‐bridge model that also accounts for cross‐bridge distortion (Fig. 1). The three states in the model are: unbound cross‐bridge state U, the pre‐power‐stroke state W and the force‐generating state S.

The electrophysiology and contractility models were coupled through the introduction of the intracellular calcium concentration from the Paci model as input of the Land model. In turn, the amount of calcium bound to troponin C in the Land model was used in the Paci electrophysiological model to update the free intracellular calcium concentration (Fig. 1). To couple the Paci and Land models, equations describing calcium buffering in the Paci model were altered to account for buffering by troponin and calmodulin. Calcium buffering alterations introduced to the model are described below (‘Methods, electro‐mechanical coupling approach’). This approach has been previously described (Timmermann et al., 2017; Levrero‐Florencio et al., 2020; Margara et al., 2021) and is illustrated above (Fig. 1).

In addition to the baseline hiPSC‐CM electromechanical model, a second model variant (Version 2, V2) was developed and tested. In V2, the maximal conductances of *I*_Na_, *I*_K1_ and *I*_CaL_ were uniformly scaled by a factor of 2.2, as Seibertz et al. (2023) reported higher *I*_Na_, *I*_K1_ and *I*_CaL_ conductances in more mature hiPSC‐CMs (Seibertz et al., 2023). This modification was introduced to better capture electrophysiological features associated with more mature hiPSC‐CMs, including increased upstroke velocity, more negative resting membrane potential and enhanced calcium influx. The V2 model was subjected to the same simulation protocols, calibration procedures and *in silico* drug assays as the baseline model, enabling a direct comparison of their predictive performance.

### Electro‐mechanical coupling approach

To establish the baseline hiPSC‐CM model and to couple the hiPSC‐CM (Paci2020) and Land contractility models, equations describing calcium buffering in the hiPSC‐CM model were altered to account for buffering by troponin and calmodulin. Briefly, the total calcium concentration change over time in the cell (${\left[Ca^{2+}\right]}_{\mathrm{total}}$) can be decomposed as the sum of intracellular free calcium (${\left[Ca^{2+}\right]}_{i}$), calcium bound to calmodulin (CMDN, ${\left[Ca^{2+}\right]}_{\mathrm{CMDN}}$), and to troponin C (TRPN, ${\left[Ca^{2+}\right]}_{\mathrm{TRPN}}$), such that

(1) $$\begin{array}{ccc} \frac{d{\left[Ca^{2+}\right]}_{\mathrm{total}}}{dt} & = & \frac{d{\left[Ca^{2+}\right]}_{i}}{dt}+\frac{d{\left[Ca^{2+}\right]}_{\mathrm{CMDN}}}{dt}\\ & & +\;\frac{d{\left[Ca^{2+}\right]}_{\mathrm{TRPN}}}{dt}\; \end{array}$$

Equivalently, changes in total calcium are described in the hiPSC‐CM model as:

(2) $$\begin{array}{ccc} & & \;\;\frac{d{\left[Ca^{2+}\right]}_{total}}{dt}={\left[Ca\right]}_{i,\;bufc}\\ & & \;\left(I_{leak}-\;I_{up}+I_{rel}-\frac{\left(I_{CaL}+I_{bCa}+I_{pCa}-2I_{NaCa}\;\right)}{2FV_{c}}C_{m}\right)\; \end{array}$$

where

*I*_leak_ calcium release by leakage

*I*_up_ calcium uptake by SR

*I*_rel_ calcium release via RyR

*I*_CaL_ L‐type Ca^2+^ current

*I*_pCa_ calcium pump current

*I*_bCa_ calcium background current

I_NaCa_ Na^+^/Ca^2+^ exchanger current

*F* Faraday's constant

*V*_c_ cytoplasmic volume

*C*_m_ membrane capacitance

${\left[Ca\right]}_{i,\;bufc}$ is total intracellular calcium that is buffered in the cytoplasm. It was re‐defined as a sum of calcium buffered by troponin C and calmodulin:

(3) $${\left[Ca^{2+}\right]}_{i,bufc}={\left[Ca^{2+}\right]}_{TRPN}+{\left[Ca^{2+}\right]}_{CMDN\;}\;$$

The initial Paci hiPSC‐CM model formula for ${\left[Ca\right]}_{i,\;\mathrm{bufc}}$ describes its dynamics:

(4) $${\left[Ca\right]}_{i,\mathrm{bufc}}=\frac{1}{1+\frac{Buf_{c}\cdot K_{\mathrm{bufc}}}{{\left({\left[Ca^{2+}\right]}_{i}+K_{\mathrm{bufc}}\right)}^{2}}\;}\;$$

Considering eqns (3) and (4) together yields:

(5) $${\left[Ca\right]}_{i,\mathrm{bufc}}=\frac{1}{1+\frac{\left[\underline{CMDN}\right]K_{CMDN}}{{\left({\left[Ca^{2+}\right]}_{i}+K_{CMDN}\right)}^{2}}+\frac{\left[\underline{TRPN}\right]K_{TRPN}}{{\left({\left[Ca^{2+}\right]}_{i}+K_{TRPN}\right)}^{2}}\;}\;$$

where $\left[\underline{CMDN}\right]$ and $\left[\underline{TRPN}\right]$ refer to the CMDN and TRPN concentrations that can bind calcium. Given that, the initial equation for calcium change over time in the cell becomes:

(6) $$\begin{array}{ccc} \frac{d{\left[Ca^{2+}\right]}_{total}}{dt} & = & \frac{d{\left[Ca^{2+}\right]}_{i}}{dt}+\frac{d{\left[Ca^{2+}\right]}_{CMDN}}{dt}+\frac{d{\left[Ca^{2+}\right]}_{TRPN}}{dt}\\ & = & \frac{d{\left[Ca^{2+}\right]}_{i}}{dt}\;\left(1+\frac{\left[\underline{CMDN}\right]K_{CMDN}}{{\left({\left[Ca^{2+}\right]}_{i}+K_{CMDN}\right)}^{2}}\right.\\ & & \left.+\;\frac{\left[\underline{TRPN}\right]K_{TRPN}}{{\left({\left[Ca^{2+}\right]}_{i}+K_{TRPN}\right)}^{2}}\right),\; \end{array}$$

yielding

(7) $$\begin{array}{ccc} & & \frac{d{\left[Ca^{2+}\right]}_{i}}{dt}={\left[Ca\right]}_{i,\;\mathrm{bufc}}\\ & & \;\left(I_{leak}-\;I_{up}+I_{rel}-\frac{\left(I_{CaL}+I_{bCa}+I_{pCa}-2I_{NaCa}\;\right)}{2FV_{c}}C_{m}\right),\; \end{array}$$

where ${\left[Ca\right]}_{i,\;\mathrm{bufc}}$ is described by eqn (5). Dynamic calcium buffering for troponin C was then implemented by defining calcium bound to troponin C (${\left[Ca^{2+}\right]}_{TRPN}$) as the fraction of troponin C units with calcium bound to its regulatory site (CaTRPN) multiplied by a constant maximum concentration of calcium ions that can bind to troponin C. The maximum concentration was derived as described below, giving ${\left[Ca^{2+}\right]}_{TRPN,max}$ = 0.7 mM. Dynamic calcium buffering by troponin C was described as:

(8) $$\frac{d{\left[Ca^{2+}\right]}_{TRPN}}{dt}={\left[Ca^{2+}\right]}_{TRPN,max}\;\frac{dCaTRPN}{dt}$$

The formulation provided by Land et al. (2017) was then adopted to describe the dynamic binding of calcium to troponin C:

(9) $$\begin{array}{ccc} & & \frac{dCaTRPN}{dt}=k_{TRPN}\\ & & \;\left({\left(\frac{{\left[Ca^{2+}\right]}_{i}}{{\left[Ca^{2+}\right]}_{T50}}\right)}^{n_{TRPN}}\left(1-CaTRPN\right)-CaTRPN\right),\; \end{array}$$

where

$k_{TRPN}$ calcium‐troponin unbinding rate

$n_{TRPN}$ the cooperativity of the calcium‐troponin C binding rate

${\left[Ca^{2+}\right]}_{T50}$ half‐activation point

The final formulation for the transient of the free intracellular calcium then becomes

(10) $$\begin{array}{ccc} & & \frac{d{\left[Ca^{2+}\right]}_{i}}{dt}={\left[Ca^{*}\right]}_{i,\;\mathrm{bufc}}\\ & & \;\left(\left(I_{leak}-\;I_{up}+I_{rel}-\frac{\left(I_{CaL}+I_{bCa}+I_{pCa}-2I_{NaCa}\;\right)}{2FV_{c}}C_{m}\right)\right.\\ & & \;-\left.\frac{d{\left[Ca^{2+}\right]}_{TRPN}}{dt}\right),\; \end{array}$$

where

(11) $${\left[\;Ca^{*}\right]}_{i,\;\mathrm{bufc}}=\frac{1}{1+\frac{\left[\underline{CMDN}\right]K_{CMDN}}{{\left({\left[Ca^{2+}\right]}_{i}+K_{CMDN}\right)}^{2}}\;}\;$$

#### Derivation of the maximum calcium buffering efficiencies

As per eqn (3), total buffered calcium in the cytosol can be described as the sum of calcium buffered by TRPN and CMDN. In the human adult cardiomyocyte Tomek, Rodriguez‐O'Hara, Rudy (ToR‐ORd) model, total buffered calcium by TRPN and CMDN equals 0.12 mM and buffered by TRPN alone equals 0.07 mM (Tomek et al., 2019). Assuming the *proportions* of maximal calcium that can be buffered by TRPN and CMDN remain the same, and assuming that *K*_m_ for both buffers are unchanged, the TRPN:CMDN ratio was established as:

$$\begin{array}{ccc} \;\left[\underline{CMDN}\right] & = & 0.05\;\mathrm{mM};\;\;\left[\underline{TRPN}\right]\\ & = & 0.07\;\mathrm{mM};\;\left[\underline{CMDN}\right]\;:\;\left[\underline{TRPN}\right]=5\;:7 \end{array}$$

Given that TRPN can buffer 1.4 times more calcium, the CMDN concentration for the hiPSC‐CM electromechanical model was found by utilizing the genetic algorithm (*ga*) function on MATLAB with default parameters. The *ga* function was used to find the lowest value of a cost function which was as outlined as:

$$\;d_{t}=d\left(\mathrm{TP},\left[217\right]\right)+d\left(\mathrm{ampl}\mathrm{itude},\left[0.152\right]\right)$$

where the distances *d* between CaT biomarkers *TP* and *amplitude* were computed and summed to obtain the total cost $d_{t}$, which was then minimized. This allowed us to find CMDN and TRPN concentrations that provide the best fit for CaTs between the hiPSC‐CM electromechanical and hiPSC‐CM electrophysiological (Paci2020) models. The values for TRPN and CMDN concentrations were [TRPN]≅0.42; [CMDN]≅0.3.

### Calibration of the hiPSC‐CM electromechanical model

The hiPSC‐CM electromechanical model was then calibrated by using experimental active tension hiPSC‐CM markers to adjust parameters in the adult human myocyte contractility model, which is explained in detail below. The re‐parameterization of the model was needed to make the model representative of the contractile behaviour observed in hiPSC‐CMs. In particular, CaT from the hiPSC‐CM electrophysiology model was different from those in the Land model to drive contraction. Therefore, the Land model parameters were re‐parameterized to achieve the physiologically active tension of hiPSC‐CMs. The calibrated Land model parameters were the Hill coefficient of cooperative activation and the tropomyosin rate constant.

Details on the fitting procedure, including the choice of model parameters to be varied, the marker ranges, the cost function and the algorithm used can be found below. The calibrated model was then used to evaluate the various physiological effects of electro‐mechanical coupling. Model outputs are summarised in Table.

A summary of the experimental data used for the model calibration is provided in Table 2. The hiPSC‐CM engineered heart tissue (EHT) data from Schwan et al. (2016, 2019) were used for calibrating the baseline version 1 model using the amplitude and the kinetic markers of active tension. Additional data were used for calibration of the model using the active tension amplitude only, since other parameters were not reported. The hiPSC‐CM data from Garg et al. (2018) were used to calibrate the second version of the model (V2) reflecting an alternative parameterization aimed at capturing calcium transient characteristics associated with more mature‐like hiPSC‐CM behaviour. A summary of the experimental data used for the model validation is provided in Table 3. This includes hiPSC‐CM action potential (AP) and calcium transient (CaT) recordings as well as active tension data acquired in the lab. For the active tension data, the measurements were performed as described before (Casini et al., 2023) using the Chiaro Nanoindenter system from Optics11 on hiPSC‐CMs on day 30 of maturation. During measurements, cells were kept at 37°C in the recording solution (NaCl 140 mM, KCl 5.5 mM, Hepes 10 mM, MgCl_2_ 1 mM, glucose 10 mM, CaCl_2_ 1.8 mM) and spherical glass tips with 3.0–3.4 µm radius and 0.033 N/m stiffness were used to indent the cell surface.

### Calibration of the model

After coupling of the models, the hiPSC‐CM electromechanical model was established. To calibrate it to the experimental active tension hiPSC‐CM data, active tension parameters were changed. Two parameters were selected for future fitting: *nTm* and *ku*. The data used for calibration were collected from hiPSC‐CM experiments only (embryonic stem cell experimental data not included), under 1 Hz pacing, at 37°C to match simulation and experimental conditions.

*Model parameters fitted in the calibration process*:

**Table:** 

	*ku*	*nTm*
Parameter meaning	The rate at which myosin binding sites on actin move from the blocked (*B*) to unbound (*U*) state	Hill coefficient of the cooperative Hill curve describing a steady‐state relation between troponin C units with calcium bound (CaTRPN) and the fraction of unblocked myosin binding sites on actin
Parameter range	0.01–2	1.5–2.5
Original value	1	5
Calibrated hiPSC‐CM electromechanical model	1.962	2.3

Parameter fitting was achieved by minimizing the cost function which includes TP, RT50, and maximum and minimum active tension, which were extracted after running simulations and computing active tension using the hiPSC‐CM electromechanical model. The cost function used here was an altered formula of the cost function used by Land et al. (2017):

$$\begin{array}{ccc} \;d_{t} & = & d\left(\mathrm{TP},\left[169,215\right]\right)+d\left(\mathrm{RT}50,\left[141,178\right]\right)\\ & & +\;10d\left(\max\left(\mathrm{Ta}\right),\left[0.21,6.5\right]\right)+25\min\left(\mathrm{Ta}\right) \end{array}$$

where the distances *d* between each biomarker and the corresponding experimental ranges were computed and summed to obtain the total cost $d_{t}$ which was then minimized. The ranges of biomarkers chosen as targets for this calibration procedure are denoted in the equation above and were based on Table 2. To minimize the cost function, the MatLab function *ga* was used. The function was altered by eliminating one term that was included in the original formulation: d (RT90, []). This was done because of the lack of availability of hiPSC‐CM studies reporting RT90. The weights in the cost function that multiply minimal (25) and maximal (10) active tension were chosen by Land et al. (2017) to prioritize good activation and relaxation, and were kept unvaried here. However, the elimination changes the relative weight of each term used in the summation. Despite this, the minimization of the cost function allowed finding parameter values such that the biomarkers of the simulations would be in line with the experimental data ranges The calibrated hiPSC‐CM electromechanical model was used for hiPSC‐CM contractile phenotype investigations *in silico*.

### *In silico* drug assay design

Two groups of compounds were selected to assess the accuracy of simulations with the hiPSC‐CM model for drug effects on contractility. Group 1 contained seven compounds selected based on the availability of experimental hiPSC‐CM contractile studies that report quantitative effects on force of contraction and calcium transient duration (CTD90). These compounds were chosen to enable direct comparison between simulated and experimental data. The compounds included: Bay‐K8644, a selective calcium channel activator (*I*_CaL_); E‐4031, an *I*_Kr_ blocker; and the multiple channel (*I*_CaL_, *I*_Kr_, *I*_Na_) blockers sunitinib, bepridil, lidocaine, nifedipine, and verapamil. Group 2 included 64 compounds where the simulated inotropic effects were compared to known clinical and experimental data for 48 drugs, and for 16 drugs, no inotropic data were available. This group was selected to evaluate the broader predictive capability of the model across a diverse range of drugs, including anti‐arrhythmics, anti‐psychotics, antibiotics, kinase inhibitors and antivirals, many of which exhibit multichannel action. The data for these drugs were acquired under different experimental and clinical conditions, from various research models, under different concentrations of the drugs used. Since most of these drugs act on a diversity of channels, prediction and interpretation of cardiotoxicity is challenging. By testing these compounds, we aimed to assess the model's ability to predict complex drug effects.

The experimental IC50 and Hill coefficients (*h*) used for the drug assays for different ion channels were considered: fast Na^+^ current (*I*_Na_), rapid/slow delayed rectified K^+^ current (*I*_Kr_/*I*_Ks_), transient outward K^+^ current (*I*_to_), L‐type Ca^2+^ current (*I*_CaL_), inward rectifier K^+^ current (*I*_K1_), and late Na^+^ current (*I*_NaL_). This was based on the availability of the data, where these inputs were successfully used *in silico* drug assays before. The data were from Passini et al. (2017), Forouzandehmehr et al. (2021) and Tomek et al. (2019).

For the compounds that were included in more than one of these ion channel datasets, multiple profiles were considered to investigate the impact of variability in drug characterization. Each IC50 and *h* set was simulated separately, resulting in more than 64 different drug trials (Table 4).

Multiple concentrations were investigated for each compound, chosen to match those used in the experimental drug assays, as well as to explore different multiples of the maximal effective free therapeutic concentration (EFTPCmax), up to 100‐fold. The EFTPCmax values were taken from the literature. The full list of compounds, together with the IC50, Hill coefficient and the EFTPCmax used for *in silico* drug trials are provided in Table 4. Simulated drug effects were considered as positive or negative inotropic when the change in peak force amplitude was greater than 10% of baseline to ensure only robust changes were registered as positive and negative. The simulations were run for multiple different doses of compounds: multiples of the EFTPCmax up to 100‐fold.

### Simulation protocols

For the comparison of simulations between the baseline hiPSC‐CM electromechanical model and the previously established hiPSCM‐CM electrophysiological model, paced cell activity was simulated. For *in silico* drug trials, simulations were run in a spontaneously beating cell mode to also investigate drug‐induced effects on the spontaneous beating frequency.

Simulations were conducted using MatLab (Mathworks Inc. Natwick, MA, USA) with the ordinary differential equation solver ode15s. For the simulations in paced mode, stimulus currents of 5 ms duration and 550 pA amplitude were used. Cell length was kept unchanged unless stated otherwise, meaning that the extension ratio (sarcomere length over sarcomere length at rest) was set to 1. For simulations in both the paced mode (1 Hz) and the non‐paced mode at 37°C, simulated data were computed in the steady state (after 800 s). Drug assay simulations were run at 37°C. Additional simulations were run in the paced mode (0.5 Hz) at 20°C for additional comparisons with the experimental dataset (see Fig. 6).

We assessed the drug‐induced changes on AP and CaT markers, as well as the occurrence of abnormalities. AP markers were extracted, including AP duration at 50 and 90% of repolarization (APD50, APD90), AP amplitude (APA) and cycle length (CL). Calcium markers extracted included CTD50 and CTD90. Active tension markers extracted included force amplitude (AT), and time to 50 and 90% relaxation (RT50 and RT90). Drug‐induced changes in those markers are presented as percentage change with drug, compared to control (no drug).

Single and multiple early afterdepolarizations (EADs) were defined as extra‐peaks greater than −55 mV in between two consecutive AP upstrokes. Repolarization failure was identified when a stable d*v*/d*t* < 0.1 V/s at a membrane potential that remained more depolarized than −40 mV was observed during the last 15 s of simulation. Irregular rhythm was identified when the difference in cycle length between two consecutive AP was greater than 150%. We also assessed quiescence, that is, when a model did not produce spontaneous APs. This was defined as a model which during the last 15 s had an average membrane potential lower than −40 mV.

### Experimental data for evaluation of the *in silico* drug trials

As the inotropic effects of some simulated drugs were previously unknown, additional experiments were performed in hiPSC‐CMs for some representative compounds to assess the changes in intracellular calcium dynamics after the addition of varying concentrations of duloxetine hydrochloride (ThermoFisher, Waltham, MA, USA) and paliperidone (Cambridge Bioscience, Cambridge, UK). The experiments comply with the ethics policies for working with cell lines. First, human induced pluripotent stem cells [hiPSCs; M398 line reprogrammed from human adult myoblasts from healthy individuals, a gift from Professor Pinset lab (Massouridès et al., 2015)], were cultured as described before (Campostrini et al., 2021; Subramaniam et al., 2023). Briefly, the cells were cultured in mTeSR Plus medium (Stemcell Technologies, Cambridge, UK) on Matrigel (8 µg/cm^2^; Corning, New York, USA)‐coated surfaces. Cells were passaged twice a week using DPBS (Gibco, Fisher Scientific, Loughborough, Leicestershire, UK) for rinsing and accutase (Sigma‐Aldrich, St Louis, MO, USA) for dissociation. The ROCK (Rho Kinase) inhibitor (StemMACS Y27632, 10 µmol/L; Miltenyi Biotech, Bergisch Gladbach, Germany) was added for the first 24 h after seeding. Differentiation into cardiomyocytes was induced in a monolayer as described previously (Campostrini et al., 2021). Differentiated human inducible pluripotent stem cell‐derived cardiac myocytes (hiPSC‐CMs) were maintained in RPMI 1640 medium with Glutamax and 25 mM Hepes (Gibco) plus B27 supplement (Gibco).

At 21 days after differentiation, seeded hiPSC‐CMs were incubated with Fura‐2 (1 µM, 15 min, 37°C). For calcium imaging, cells were kept in an imaging buffer (NaCl 140 mmol/L, KCl 3 mmol/L, Hepes 10 mmol/L, glucose 15 mmol/L, CaCl_2_ 2 mmol/L, MgCl_2_ 2 mmol/L, pH 7.4) and imaged on an Olympus IX71 inverted microscope using a PlanApoN, ×40, oil immersion objective (NA 1.4, 0.17/field number 26.5, Olympus, UK) using IonOptix hardware and software, at 0.5 Hz pacing conditions or in spontaneously beating modes at room temperature. Data were analysed and plotted using MatLab (Mathworks Inc., Natwick, MA, USA).

*In silico* drug simulation results were compared to experimental data. All experimental results are presented as percentage change with respect to baseline.

## Results

### Development, calibration and experimental evaluation of the hiPSC‐CM electromechanical model

**Figure 2:**
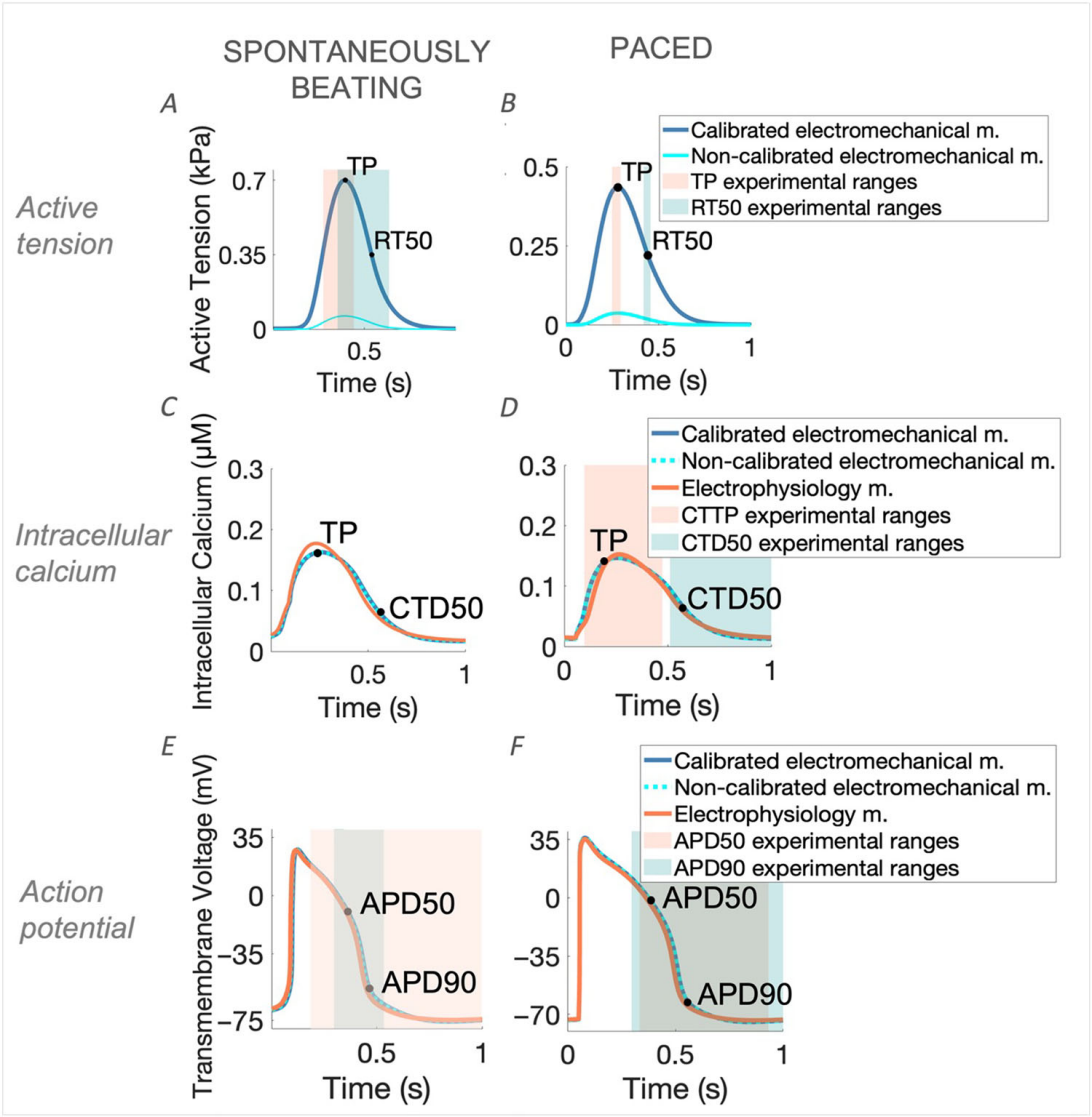
Comparison of the electro‐mechanical and electrophysiology‐only hiPSC‐CM models and available experimental wet‐lab hiPSC‐CM data In cases where calibrated (dark blue) and non‐calibrated (bright cyan) model simulations result in the same outputs, the line is visualized as dashed. Experimental ranges for markers are represented as shaded areas. In spontaneously beating cells, simulated *A*, active tension; *C*, calcium transient; and *E*, action potential, before and after electromechanical model calibration. In *C* and E, the outputs are also compared to the electrophysiology‐only model simulations. In paced (1 Hz) cells, simulated *B*, active tension; *D*, calcium transient; and *F*, action potential, before and after electromechanical model calibration. In *D* and *F*, the outputs are also compared to the electrophysiology‐only model simulations. Here TP = time to peak tension, RT50 = time to 50% relaxation, APD50 = time to 50% repolarization, and APD90 = time to 90% repolarization.

Figure 2 shows the comparison of simulations with the newly developed hiPSC‐CM electromechanical model and the electrophysiology‐only model (Paci et al., 2020). Electro‐mechanical coupling does not affect the normal kinetics of calcium dynamics and action potentials. However, the non‐calibrated electromechanical model did not produce active tension markers in line with experimental data.

The calibration of model parameters largely improved the contractility performance of the electromechanical model. In particular, the active tension amplitude increased from 0.04 to 0.4 kPa (wet‐lab experimental data range: 0.21–6.5 kPa) in simulated paced cells (1 Hz) after calibration. The time to peak tension remained largely the same, at 201 ms (experimental data range: 169–215 ms), and the relaxation time (RT50) changed from 158 to 164 ms (experimental data range: 141–178 ms). After calibration, the simulated active tension, calcium transient and action potential markers were in line with experimental data (Table 1, Figs 1 and 2). Similar results were also observed in the spontaneously beating mode (Fig. 2, Table 1).

**Table 1:** Active tension, CaT and AP biomarker values generated from simulations with the Paci, Land, ToR‐ORd‐Land, and uncalibrated electromechanical hiPSC‐CM models with original parameter values and calibrated electromechanical hiPSC‐CM models

Biomarkers	Land	ToR‐ORd‐Land	Paci	Uncalibrated el mechanical hiPSC‐CM	Calibrated el mechanical hiPSC‐CM (V1)	Calibrated el mechanical hiPSC‐CM (V2)	Experimental ranges^b^, ^c^	Paci	Uncalibrated el mechanical hiPSC‐CM	Calibrated el mechanical hiPSC‐CM (V1)	Calibrated el mechanical hiPSC‐CM (V2)	Experimental ranges^b^, ^c^	Paci‐Rice^a^
		Paced						Spontaneously beating					
Active tension (AT)													
TP, ms	175	169	N/A	201.5	201	169	169–215	N/A	219.7	214	207	63.7–232 *	
RT50, ms	121	116	N/A	158	164	177	141–178	N/A	143	147	142	69.2–182.4 *	161^a^
RT90, ms	234	227	N/A	286	329	306		N/A	258	287	267		
Max AT, kPa	51.2	24	N/A	0.04	0.44	18.4	0.21–6.5	N/A	0.062	0.7	20		0.055^a^
Min AT, kPa	0.0078	0.075	N/A	0	0	0	0–0.58	N/A	0	0	0		
Calcium transient													
CTTP, ms	124	42.4	207.3	200.4	201	173	97–474^b^; 163.7–326.3^c^	218	232	232	212.7		
CTD50+TP, ms	337	201.9	369.6	427	427.5	428.5	511–1168	345.9	387.7	387.6	383.6		
CTD90+TP, ms	567	355.4	613.3	631.3	632.7	658.7	310–1803	550.4	576	576	624		
Amplitude, µM	0.6	0.475	0.152	0.147	0.147	0.37	0.16^b^; 0.376– 0.674^c^	0.177	0.163	0.163	0.379		
Action potential													
APD50, ms	N/A	219.5	393.3	408	408	346	333–932	300.5	303.5	303.5	261	175–1059	
APD90, ms	N/A	273.5	483.5	505.2	505	524	296–1398	390.4	397.7	397.5	471	296–534	403^a^
Amplitude, mV	N/A	121.63	108.8	110.6	110.6	134	98–112	102	103.3	103.3	115	98–110	103^a^

Experimental ranges are based on hiPSC‐CM data established from a literature search (Tables 2 and 3), and acquired experimentally in the lab for this study, as indicated with an asterisk. The Land model describes mechanical adult ventricular cardiomyocyte behaviour, ToR‐ORd‐Land describes electromechanical adult cardiomyocyte behaviour, Paci describes electrophysiology of hiPSC‐CM, the electromechanical hiPSC‐CM model is the newly established hiPSC‐CM electromechanical model, and Paci‐Rice is an iPSC‐CM electromechanical model based on a previously developed contractile model formulation (Rice et al., 2008), which has been adapted and reparameterised using human iPSC‐CM experimental data (Forouzandehmehr et al., 2021). TP: time to peak; RT50: time to 50% relaxation; RT90: time to 90% relaxation; AT: active tension; CTTP: CaT time to peak; CTD50: CaT duration at 50% decay; CTD90: CaT duration at 90% decay; APD50: AP duration at 50% repolarization; APD90: AP duration at 90% repolarization. [Correction made on 22 May 2026, after first online publication: the preceding sentence has been updated to clarify that the electromechanical hiPSC‐CM model of Forouzandehmehr et al. (2021) relies on an established contractile formulation that is adapted using hiPSC‐derived measurements.]

^a^ Forouzandehmehr et al (2021).

^b^ Data sources are provided in Tables 2 and 3.

^c^ Data by Garg et al. (2018) have been acquired from cells paced at 0.5 Hz.

**Table 2:** Summary of the experimental data for model calibration from hiPSC‐CMs only [human embryonic stem cell (hESC) data not included]

Reference	Methods	Measurement protocol	Cell maturity	Data type	Biomarkers
Clark et al. (2021)	‐ ETH derived from T cells from an adult female patient with HCM, as well as a genome‐edited derivative line	‐Force dynamics assessed as in Schwan et al. (2016) ‐ ETHs were mounted on a testing setup consisting of a force transducer and a micromanipulator enabling muscle stretching, and paced at 1 Hz, 35 °C	Experiments performed at day 32 post differentiation (day 19 post‐differentiation seeded onto scaffolds to generate ETH)	RT50 for AT, active force generated by gradual stretch (to 10% stretch)	RT50: 158 ± 12 ms
Ballan et al. (2020)	‐ hiPSC line from dermal fibroblasts ‐reprogrammed by retroviral vectors encoding SOX2, KLF4 and OCT4 ‐differentiated by the growth factors, EB‐based process	‐single‐cell force measurement system (2 micromanipulators, an optical force transducer and a piezoelectric length controller) ‐ hPSC‐CMs were attached to the coated fibres at cell edges, separated from the substrate and lifted, and paced at 1 Hz	Experiments performed at day 22 post differentiation	Mean AT was measured at baseline cell length with subsequent stretching reaching 40% stretch	Mean AT at baseline length: 0.51 ± 0.06 mN/mm^2^; maximal AT after stretch: 1.24 ± 0.08 mN/mm^2^. Peak total tension: 1.82 ± 0.25 mN/mm^2^.
Kensah et al. (2013)	‐hiPSC differentiation and EB formation induced by antibiotic‐mediated selection of CMs using G418 to generate human PSC‐derived bioartificial cardiac tissues on a Matrigel and collagen scaffold	‐constructs were placed into a custom‐made bioreactor for force measurements ‐tissue constructs were subject to stress ‐paced at 1 Hz, RT	Measurements performed at day 14–21 post differentiation	Contraction forces at *L*_max_; forces after stretching (5–10%)	AT: 4.4 mN/mm^2^. Contraction force: 0.62 ± 0.03 mN on d14; 0.75 ± 0.06 mN on d21. Force after cyclical stretching: 0.77 ± 0.07 mN (*n* = 5); after static: 0.97 ± 0.08 mN (*n* = 8).
Sewanan et al. (2019)	‐hiPSCs from T cells, differentiated by modulating Wnt signalling ‐hiPSC‐CMs used for ETH generation	‐Force dynamics assessed as in Schwan et al. (2016) ‐ ETHs were mounted on a testing setup consisting of a force transducer and a micromanipulator enabling muscle stretching, and paced at 1 Hz, at 35 °C	Measurements performed at day 15 post differentiation	Active force generated by gradual stretch (0.015 mm/s to 10% stretch)	TP: 211 ± 4 ms; RT50: 143 ± 2 ms; peak twitch force: 0.05 ± 0.01 mN; peak stress: 0.22 ± 0.1 mN/mm^2^.
Schwan et al. (2016)	‐hiPSC‐CMs from peripheral blood mononuclear cells ‐differentiated using a Sendai virus and with a monolayer differentiation protocol ‐cells seeded on scaffolds forming ETHs.	‐clips holding EHT were ejected from the frame and picked up by motorized micromanipulators with claw‐like extensions, leaving the muscle suspended between an anchoring attachment claw and a force transducer on a second claw; EHTs were immersed in a perfusion bath, paced at 1 Hz, at 35 °C	Measurements performed at day 23 post differentiation	AT at 8% stretch	Average (*n* = 8) peak stress: 2.2 ± 0.76 mN/mm^2^; maximum peak stress recorded: 6.5 mN/mm^2^; TP: 181 ± 12 ms; RT50: 168 ± 10 ms
Ruan et al. (2016)	‐hiPSCs from a lung fibroblast line ‐differentiated by monolayer‐based differentiation protocol ‐hiPSC‐CMs used for cardiac‐like organoid (3D scaffold) generation	‐constructs were dissected into sections and suspended on stainless steel hooks attached to a force transducer and a length controller in a chamber perfused with Tyrode solution, paced at 2 Hz, 37°C.	Measurements performed at day 14–21 post differentiation	AT determined by stretching the structure in small increments (4% length) to 125% of initial length	Twitch amplitude: 0.37 mN/mm^2^

TP: time to peak; RT50: time to 50% decay; APD50 and APD90: action potential (AP) duration at 50% and 90% repolarization, respectively; AT: active tension; RT: room temperature; EBs: embryoid bodies; CMs: cardiomyocytes; EHTs: engineered human tissues; *L*_max_: cell length at which maximum twitch force is reached; *n*: sample size. The data were collected from hiPSC‐CM‐derived EBs, EHTs and other constructs. Data reported as mean ± SD.

**Table 3:** Summary of the experimental data used for model validation

Reference	Tissue preparation	Measurement protocol	Cell maturity	Data type	Biomarkers
Garg et al. (2018)	‐hiPSC‐CMs from peripheral blood mononuclear cells (*n* = 37) ‐differentiated with Sendai virus and a 2D monolayer differentiation protocol	‐recordings made using patch clamping at 36–37°C, paced at 0.5 Hz.	Measurements performed at day 30–50 post differentiation	CaT, AP	CaT: TP: 245.0 ± 81.3 ms; RT50: 450 ± 100 ms; RT95: 990 ± 50 ms; AP amplitude: 111.26 ± 0.99mV; APD90: 367.17 ± 19.62 ms; APD50: 308.33 ± 17.09 ms
Rao et al. (2013)	‐hiPSC‐CMs from iCell Cardiomyocytes™ on structured and non‐structured substrates	‐AP was measured using a current clamp system ‐CaT was measured under field stimulation using an external pacing generator ‐ paced at 0.5 and 1 Hz at 37 °C	Measurements performed at day 14 post hiPSC‐CM seeding	CaT Spontaneous AP	Non‐structured at 1 Hz, CaT: TP: 195 ± 10 ms; RT50: 285 ± 20 ms; RT90: 670 ± 20 ms; APD: non‐structured: TP: 57 ± 6 ms; RT90: 550 ± 10 ms Structured: TP: 55 ± 6 ms; RT90: 548 ± 12 ms
Cordeiro et al. (2013)	‐hiPSC cell line reprogrammed with Oct4, Nanog, Lin28 and Sox2 ‐differentiated using media supplemented with BMP4, Activin A, bFGF, VEGF and DKK‐1	‐ microelectrodes connected to an Axoclamp 2A amplifier were used to record APs from spontaneously beating clusters superfused with a Hepes‐buffered Tyrode solution at 37 °C	Measurements performed day 11–19 post differentiation	AP	Cells w/o a notch (56% ventricular‐ and 44% atrial‐like): APD90: 277.3 ± 9.0; APD50: 211.0 ± 8.2 ms; cycle length: 1494 ± 144 ms; amplitude: 101.8 ± 0.9 mV. Cells with a notch (17% ventricular‐ and 83% atrial‐like): APD90: 296.9 ± 87.5 ms; APD50: 210.9 ± 69.3; cycle length: 1632 ± 566; AP amplitude: 109.2 ± 1.7 mV.
Fatima et al. (2011)	‐hiPSCs from skin fibroblasts reprogrammed with OCT3/4, SOX2, KLF4 and c‐MYC ‐diff. induced by co‐culturing hiPSCs with murine visceral endoderm‐like cell line END2	‐ whole‐cell current clamp technique performed with an EPC‐9 amplifier was used for AP measurements at 37 °C	Measurements performed at day 20–30 post differentiation	AP	APD90 for ventricular‐like cells: 297.70 ± 60.53 ms; APD50 175.45 ± 43.25 ms
Honda et al. (2011)	‐hiPSCs from fibroblasts ‐reprogrammed with retrovirus encoding Oct3/4, SOX2, KLF4 and c‐Myc, EBs formed	‐after colony dissociation, single cells were patch‐clamped to measure AP at RT	Measurements performed at day 28–44 post differentiation	AP, early afterdepolarizations (EAD)	APD90: 429 ± 34 ms, APD50: 357 ± 29 ms, amplitude: 101.5 ± 2 mV
Ma et al. (2011)	‐hiPSCs from fibroblast reprogrammed by retroviral vectors with SOX7, OCT4, nanog and lin28	‐ APs were recorded from single cardiomyocytes by the whole cell perforated patch‐clamp technique at 35–37°C, paced at 1 Hz	Measurements performed at day 10–21 post differentiation	AP, EAD	APD90: 414.7 ± 21.8 ms

CaT: calcium transient; AP: action potential; bpm: beats per minute.

**Table 4:** Summary of compounds, together with the IC50, Hill coefficient and EFTPCmax used for *in silico* drug trials as well as experimental and clinical data used for comparison, together with the data sources

				**IC50 (Hill coefficient)**									
**Compound list**		**Description**	**Inotropic effect (clinicalal & experimental data)**	*I*_Na_	*I*_Kr_	*I*_CaL_	*I*_NaL_	*I*_Ks_	*I*_to_	*I*_K1_	**EFTPCmax (µM)**	**hiPSC‐CM force amplitude**	**References**
**1**	**Amiodarone I**	Anti‐arrhythmic Class III	negative	15.9 (0.97)	0.86(1.09)	1.9 (0.69)					0.155	Unknown	Kramer et al., 2013; Crumb et al., 2016; Giardina et al., 2023
	**Amiodarone II**			4.577 (0.7)	0.941 (0.6)	1.281 (0.6)	9.423 (0.4)		3.758 (0.4)				
**2**	**Astemizole**	H1‐receptor antagonist, an anti‐allergic agent	no effect	3 (1.95)	0.004 (0.78)	1.1 (1.66)					0.0003	Unknown	Kramer et al., 2013; Sugiyama et al., 1997
**3**	**BaCl2**	Potassium channel blocker	positive		257 (1)					4.5 (1)	1	Increase	Passini et al., 2017; Forouzandehmehr et al., 2021; Schram et al., 2003; Nguyen et al., 2017
**4**	**Bay‐K8644**	Calcium channel activator	positive			17.3 (1.25)						Increase	Ruan et al., 2016; Saleem et al., 2020
**5**	**Bepridil**	Calcium channel blocker	negative	2.929 (1.2)	0.149 (0.9)	2.808 (0.6)	1.814 (1.4)				0.035	Decrease	Crumb et al., 2016; Paci et al., 2020; Blinova et al., 2018
**6**	**Ceftriaxone**	Antibiotic	unknown		445.7 (1)	153.8 (1)					23.17	Unknown	Kramer et al., 2013
**7**	**Chloropromazine I**	Antipsychotic	negative		1.5 (1.4)	3.4 (1.73)					0.038	Unknown	Kramer et al., 2013; Crumb et al., 2016; Juriavicius et al., 1989
	**Chloropromazine II**				1.118 (0.9)	8.192 (0.8)	4.56 (0.9)			9.27 (0.7)			
**8**	**Cilostazol**	Anticoagulant (anti‐platelet)	positive		13.8 (0.91)	91.2 (1)					0.128	Unknown	Kramer et al., 2013; Schrör et al., 2002
**9**	**Cisapride I**	Gastro‐prokinetic	negative		0.02 (1.04)	11.8 (1)					0.003	Decrease	Kramer et al., 2013; Crumb et al., 2016; Keller et al., 2018
	**Cisapride II**				0.012 (1.3)								
**10**	**Clozapine**	Anti‐psychotic	negative	15.1 (1.14)	2.3 (0.97)	3.6 (1)					0.071	Unknown	Kramer et al., 2013; Abdel‐Wahab et al., 2014
**11**	**Dasatinib**	Tyrosine kinase inhibitor	negative	76.3 (1.43)	24.5 (1.16)	81.1 (1)					0.041	Unknown	Kramer et al., 2013; Motokawa et al., 2022
**12**	**Diazepam**	Benzodiazepine	positive	306.4 (1)	53.2 (1.07)	30.5 (0.89)					0.029	Unknown	Kramer et al., 2013; Martinez et al., 1995
**13**	**Diltiazem**	Calcium channel blocker	negative	22.4 (1.29)	13.2 (1.16)	0.76 (1.14)					0.1275	Decrease	Kramer et al., 2013; Talreja et al., 2023; Zeng et al., 2018
**14**	**Disopyramide**	Anti‐arrhythmic Class Ia	negative	168.4 (1.09)	14.4 (0.91)	1036.7 (1)					0.742	Unknown	Kramer et al., 2013; Lan et al., 2013
**15**	**Dofetilide I**	Anti‐arrhythmic Class III	no effect	31.9 (0.54)	0.013 (1.56)	201 (1)		135 (1)	300 (1)		0.0021	Decrease	Kramer et al., 2013; Passini et al., 2017; Crumb et al., 2016; Blair & Pruitt, 2020; Kijlstra et al., 2015
	**Dofetilide II**			162.1 (1)	0.03 (1.2)	26.7 (1)							
	**Dofetilide III**				0.001 (0.6)								
**16**	**Donepezil**	Acetylcholinesterase inhibitor	negative	38.5 (1)	0.7 (0.98)	34.3 (0.83)					0.007	Unknown	Kramer et al., 2013; Page et al., 2016
**17**	**Droperidol**	Butyrophenone anti‐psychotic drug	negative	22.7 (1.24)	0.06 (1.1)	7.6 (1.16)					0.016	Unknown	Kramer et al., 2013; Shiga et al., 2005
**18**	**Duloxetine**	Serotonin‐norepinephrine reuptake inhibitor	unknown	5.1 (1.66)	3.8 (1.39)	2.8 (1.41)					0.016	Unknown	Kramer et al., 2013
**19**	**E‐4031**	Anti‐arrhythmic Class III	negative		0.017 (0.7)							Decrease	Kramer et al., 2013; Keung et al., 2019
**20**	**Flecainide I**	Anti‐arrhythmic Class Ic	negative	3.19 (0.71)	1.64 (0.81)	27.1 (0.97)					0.752	Decrease	Kramer et al., 2013; Passini et al., 2017; Crumb et al., 2016; Yang et al., 2021
	**Flecainide II**			6.2 (1.14)	1.5 (0.88)	27.1 (0.97)							
	**Flecainide III**			6.677 (1.9)	0.692 (0.8)	25.599 (1.4)	18.87 (0.6)		9.266 (0.7)				
**21**	**Halofantrine**	Anti‐malarial	unknown	331.2 (1)	0.38 (1.31)	1.9 (0.99)					0.172	Unknown	Kramer et al., 2013
**22**	**Haloperidol**	Antip‐sychotic	negative	4.3 (1.58)	0.04 (1.18)	1.3 (1.38)					0.004	Decrease	Kramer et al., 2013; Hatip‐Al‐Khatib et al., 2002; Zhao et al., 2017
**23**	**HMR 1556**	*I*_Ks_ channel blocker	no effect					0.214 (1.2)				No Effect	Kramer et al., 2013; Mannhardt et al., 2016
**24**	**Ibutilide**	Anti‐arrhythmic Class III	negative	42.5 (1.03)	0.018 (1.53)	62.5 (1.16)					0.14	Unknown	Kramer et al., 2013; Kaufman et al., 1999
**25**	**Lamivudine**	Nucleoside reverse transcriptase inhibitor	unknown	1571.4 (1)	2054 (1)	54.2 (0.89)					19.54	Unknown	Kramer et al., 2013
**26**	**Lidocaine I**	Anti‐arrhythmic Class Ib	negative	44 (0.94)	300 (1)						2.6	Decrease	Passini et al., 2017; Crumb et al., 2016; Mannhardt et al., 2016; Wilson et al., 1993
	**Lidocaine II**			44 (0.94)	300 (1)		10.79 (1.3)						
**27**	**Linezolid**	Antibiotic	unknown	2644.5 (1)	1147.2 (1)	105.4 (0.94)					59.11	Unknown	Kramer et al., 2013
**28**	**Loratadine**	H1‐anti‐histamine	no effect	28.9 (1.64)	6.1 (1.44)	11.4 (1.38)					0.0004	Unknown	Kramer et al., 2013; Davila et al., 2006
**29**	**Metronidazole**	Antibiotic	negative	2073.2 (1)	1340.2 (1)	177.9 (0.66)					187	Unknown	Kramer et al., 2013; Essien et al., 1985
**30**	**Mexiletine I**	Antiarrhythmic Class Ib	negative	49.7 (0.94)	69.4 (1.11)	203 (0.75)		32.6 (0.92)	367 (0.91)		2.5	Unknown	Passini et al., 2017; Crumb et al., 2016; Manolis et al., 1990
	**Mexiletine II**			49.7 (0.94)	69.4 (1.11)	203 (0.75)	8.957 (1.4)	32.6 (0.92)	367 (0.91)				
**31**	**Mibefradil I**	Calcium channel blocker	negative	5.6 (1.53)	1.7 (1.38)	0.51 (1.44)					0.012	Unknown	Kramer et al., 2013; Crumb et al., 2016; Mulder et al., 1997
	**Mibefradil II**			5.866 (1)	0.307 (0.9)	0.652 (1.1)	3.628 (1.2)						
**32**	**Mitoxantrone**	Antineoplastic	positive	93.5 (1.05)	539.4 (1)	22.5 (0.64)					0.225	Unknown	Kramer et al., 2013; Wang et al., 2000
**33**	**Moxifloxacin I**	Antibiotic	no effect	1563 (1)	79 (1)	173 (1)					10.96	Unknown	Kramer et al., 2013; Passini et al., 2017; Crumb et al., 2016; Mason et al., 2017
	**Moxifloxacin II**			1112 (1)	86.2 (0.94)	173 (1)							
	**Moxifloxacin III**				93.041 (0.6)		382.337 (1.1)	50.321 (1)					
**34**	**Nifedipine**	Calcium channel blocker	negative	88.5 (0.71)	44 (0.8)	0.012 (1.02)					0.008	Decrease	Kramer et al., 2013; Zhao et al., 2017; McDonald et al., 1994
**35**	**Nilotinib I**	Tyrosine kinase inhibitor	negative	13.3 (2.11)	1 (0.96)	17.5 (1)					0.172	Unknown	Kramer et al., 2013; Crumb et al., 2016
	**Nilotinib II**				0.091 (0.8)		4.706 (1.9)						
**36**	**Nimodipine**	Calcium channel blocker	negative		45.6 (1)	0.139 (0.63)					0.001	Unknown	Passini et al., 2017; Xu & Lipscombe, 2001; Colucci et al., 1985
**37**	**Nisoldipine**	Calcium channel blocker	negative	45 (0.72)	49.3 (0.84)	0.009 (0.71)	61.7 (0.71)	52.8 (1.03)			0.0001	Unknown	Passini et al., 2017; Tumas et al., 1985
**38**	**Nitrendipine**	Calcium channel blocker	negative	21.6 (1.25)	24.6 (0.82)	0.025 (0.78)					0.003	Unknown	Kramer et al., 2013; Low et al., 1982
**39**	**Paliperidone**	Anti‐psychotic	unknown	109 (1.33)	0.78 (1.01)	193.9 (1)					0.069	Unknown	Kramer et al., 2013
**40**	**Paroxetine**	Selective serotonin reuptake inhibitor	positive	9.8 (1.34)	1.9 (1.26)	3.9 (1.39)					0.014	Unknown	Kramer et al., 2013; Lund et al., 1982
**41**	**Pentobarbital**	Barbiturate	negative	2686 (1)	1433.9 (1)	299 (1.38)					5.171	Unknown	Kramer et al., 2013; Chiba et al., 1976
**42**	**Phenytoin**	Anti‐colvusant	negative	72.4 (1.06)	147 (1)	21.9 (0.99)					4.36	Unknown	Kramer et al., 2013; Kennedy et al., 1971
**43**	**Pimozide**	Anti‐psychotic	unknown	1.1 (1.05)	0.04 (1.16)	0.24 (1.49)					0.0005	Unknown	Kramer et al., 2013
**44**	**Piperacillin**	Antibiotic	unknown	2433.8 (1)	3405.1 (1)	1226 (1)					114	Unknown	Kramer et al., 2013
**45**	**Primidone**	Anti‐convulsant	unknown	640 (1)	3360 (1)						20.6	Unknown	Passini et al., 2017
**46**	**Procainamide**	Anti‐arrhythmic Class Ia	negative	746.6 (1)	272.4 (1)	389.5 (0.83)					54.18	Unknown	Kramer et al., 2013; Gottlieb et al., 1990
**47**	**Quinidine**	Anti‐arrhythmic Class Ia	negative	14.6 (1.22)	0.72 (1.06)	6.4 (0.68)					3.237	Decrease	Kramer et al., 2013; Crumb et al., 2016; Zhao et al., 2017; Chiba et al., 1976
	**Quinidine**				0.343 (1)			4.899 (1.4)	3.487 (1.3)				
**48**	**Raltegravir**	Anti‐retroviral	unknown	824.2 (1)	782.8 (1)	246.7 (1)					7	Unknown	Kramer et al., 2013
**49**	**Ranolazine**	Anti‐anginal	negative	30.2 (0.8)	10.9 (0.9)	172 (0.6)	5.9 (1)				1.95	Decrease	Kramer et al., 2013; Passini et al., 2017; Antzelevitch et al., 2004; Mannhardt et al., 2016; Maier et al., 2013
	**Ranolazine**				6.49 (0.8)		7.887 (0.9)						
**50**	**Ribavirin**	Antiviral	unknown	2997.5 (1)	967 (1)	622.5 (1)					27.88	Unknown	Kramer et al., 2013
**51**	**Risperidone**	Anti‐psychotic	positive	43.3 (0.98)	0.26 (0.99)	34.2 (0.79)					0.002	Unknown	Kramer et al., 2013; Nunoi et al., 2020
**52**	**Saquinavir I**	Anti‐retroviral	unknown	12.1 (2.34)	16.9 (1.72)	1.9 (1.15)					0.4172	Unknown	Kramer et al., 2013; Crumb et al., 2016
	**Saquinavir II**			15.568 (1.2)	3.477 (1)	3.161 (1.4)	7.088 (1.2)						
**53**	**Sertindole I**	Anti‐psychotic	unknown	6.9 (1.19)	0.033 (1.25)	6.3 (1.29)					0.002	Unknown	Kramer et al., Crumb et al., 2016
	**Sertindole II**				0.011 (0.8)								
**54**	**Sitagliptin**	Anti‐diabetic	no effect	1220.8 (1)	174.7 (1)	147.1 (1)					0.442	Unknown	Kramer et al., 2013; Lenski et al., 2011
**55**	**Solifenacin**	Anti‐muscarinic	negative	1.5 (1.32)	0.28 (0.9)	4.3 (1.47)					0.003	Unknown	Kramer et al., 2013; Michel et al., 2008
**56**	**Sotalol I**	Anti‐arrhythmic Class III, Beta blocker	negative	7013.9 (1)	111.4 (0.73)	193.3 (1)					14.69	Decrease	Kramer et al., 2013; Crumb et al., 2016; Holubarsch et al., 1995; Keung et al., 2019
	**Sotalol II**				86.369 (0.9)								
**57**	**Sparfloxacin I**	Antibiotic	unknown	1465 (1)	17.7 (0.99)	88.8 (1)					1.766	Unknown	Kramer et al., 2013; Passini et al., 2017
	**Sparfloxacin II**			2555 (1)	22.1 (0.93)	88.8 (1)						Unknown	
**58**	**Sunitinib**	Tyrosine kinase inhibitor	negative	16.5 (1.22)	1.2 (1)	33.4 (1.09)					0.013	Unknown	Kramer et al., 2013; Rainer et al., 2012
**59**	**Telbivudine**	Antiviral	unknown	1095.2 (1)	422.7 (1)	713.9 (1)					19.72	Unknown	Kramer et al., 2013
**60**	**Terfenadine I**	Antihistamine	negative	2(1.81)	0.05 (1.15)	0.93 (1.8)					0.009	Unknown	Kramer et al., 2013; Passini et al., 2017; Batey et al., 2002
	**Terfenadine I**				0.01 (0.6)	0.7 (0.7)							
**61**	**Terodiline**	Potassium channel blocker	negative	7.4 (1.23)	0.65 (1.02)	4.8 (1.01)					0.145	Unknown	Kramer et al., 2013
**62**	**Thioridazine**	Anti‐psychotic	negative	1.4 (1.11)	0.5 (0.98)	3.5 (1.35)					0.98	Unknown	Kramer et al., 2013; Refsum et al., 1978
**63**	**Verapamil I**	Anti‐arrhythmic Class IV	negative	32.5 (1.33)	0.25 (0.89)	0.2 (0.8)					0.088	Decrease	Kramer et al., 2013; Crumb et al., 2016; Obejero‐Paz et al., 2015; Mannhardt et al., 2016
	**Verapamil II**			7.2 (0.95)	0.83 (1.17)	0.1 (0.7)	6.1 (1.24)	65.6 (0.92)		9.03(1)			
	**Verapamil III**				0.499(1.1)	0.202(1.1)							
**64**	**Voriconazole**	Antifungal	unknown	1550.5 (1)	490.9 (1)	414.2 (1)					7.563	Unknown	Kramer et al., 2013

In addition to the baseline version 1 model, all simulations were also performed using an alternative model variant (V2) as described in the Methods. The resulting action potential, calcium transient and active tension waveforms were similar in morphology to version 1, and the corresponding biomarkers are reported in Table 1.

### Simulated drug‐induced inotropic effects using the hiPSC‐CM electromechanical model agree with experimental hiPSC‐CM data

As a validation process, we then compared the drug‐induced inotropic effects in hiPSC‐CM electromechanical simulations with experimental hiPSC‐CM contractility and calcium data, which were not used in model development and calibration. Experimental recordings of calcium transient durations and inotropic responses were considered for the first group of drugs (Group 1, seven reference compounds, listed in Table 5) with varied actions on ion channels (including single and multichannel blockers).

**Table 5:** Simulation of calcium transients and active tension changes induced by seven reference compounds in group 1 compared to experimental data

	Bay‐K8644	Bepridil	E‐4031	Lidocaine	Nifedipine	Sunitinib	Verapamil
Calcium transient duration at 90% or repolarization (CTD90)							
Experimental data		↑	EADs		↓		↓
Simulation data	↑	↑	Repolarization failure	↓	↓	↑	↓
Inotropy (force)							
Experimental data	↑	↓	↓	↓	↓	↓	↓
Simulation data	↑	↓	↓	↓	↓	↓	↓

Simulated force amplitude and calcium transient duration at 90% depolarization (CTD90) were compared with the corresponding experimental data from different hiPSC‐CM studies (Blinova et al., 2018; Cohen et al., 2011; Keung et al., 2019; Mannhardt et al., 2017, 2020; Ruan et al., 2016). Here EADs refer to early after depolarizations.

For Bay‐K8644, a selective calcium channel activator (*I*_CaL_), simulations showed an increase in force, which had also been observed experimentally (Cohen et al., 2011; Ruan et al., 2016). For E‐4031, which was simulated as a 70% *I*_Kr_ block at 1 µM, repolarization failure was observed in some cases in hiPSC‐CM experiments (Keung et al., 2019), consistent with simulation results. For the multiple channel blockers bepridil, lidocaine, nifedipine, sunitinib and verapamil, the simulation results show a decrease in force, in good general agreement with the experimental data (Blinova et al., 2018; Cohen et al., 2011; Keung et al., 2019; Mannhardt et al., 2017, 2020; Ruan et al., 2016).

Despite the minimal input information provided by standard recordings in drug development (IC50 and Hill coefficients), the simulations revealed a good qualitative agreement with experimental data, supporting their credibility to capture drug‐specific responses (Table 5).

All drug simulations were also repeated using the alternative V2 model variant. All the outputs were consistent with those obtained using the baseline version 1 model.

### Simulated drug‐induced inotropic effects in hiPSC‐CMs are in agreement with clinical and adult cell experimental data

In addition to available hiPSC‐CM contractility experiments, we expanded the comparisons to include clinical reports and isolated cardiac cell experiments. Group 2 consisted of 64 compounds, of which 48 compounds had been previously characterized clinically or experimentally for their effects on active tension. This group included both selective channel blockers and multichannel blockers, providing a broader dataset to evaluate the model's predictive capability across diverse drug mechanisms.

**Figure 3:**
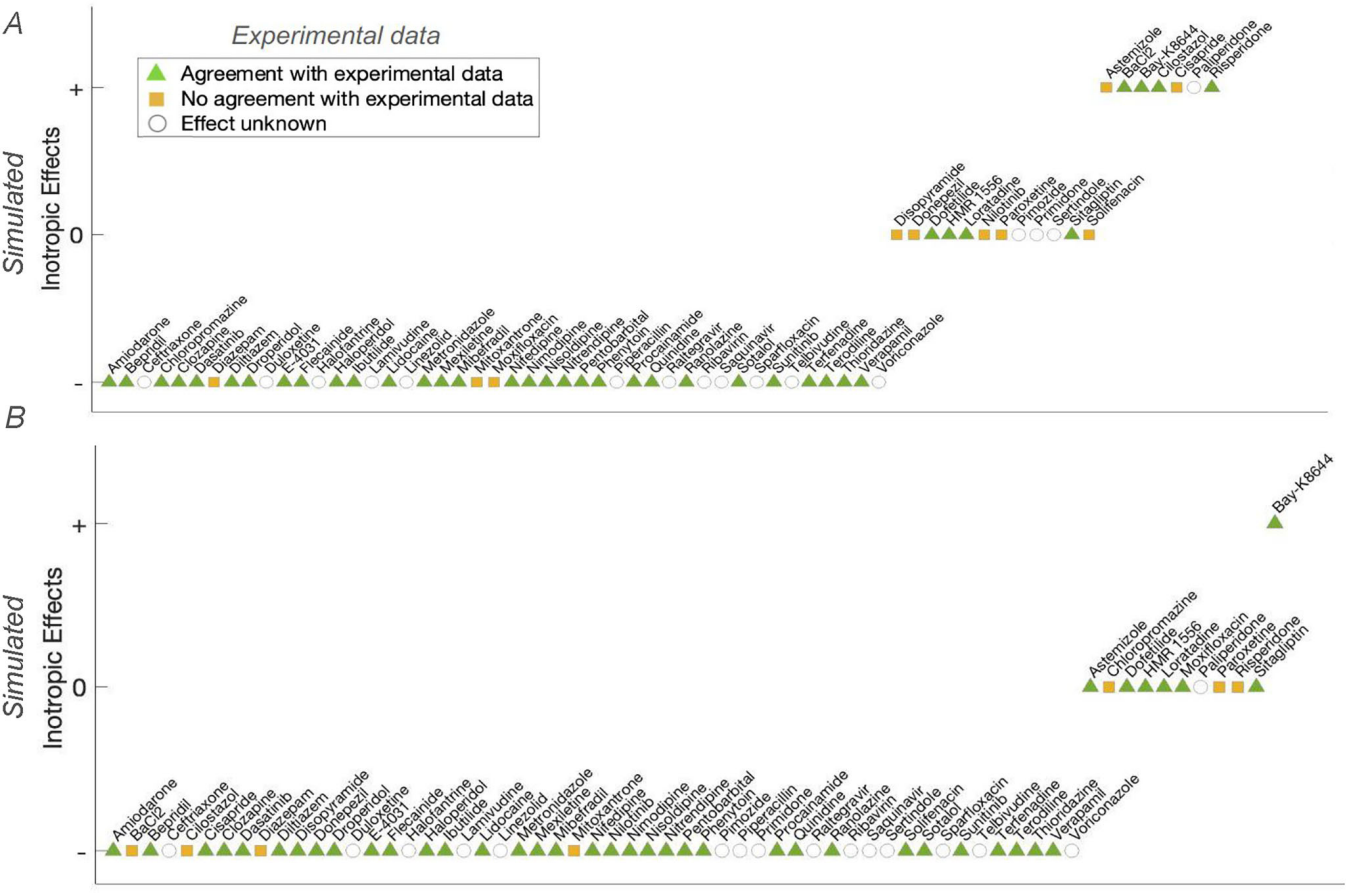
Inotropic effects for 64 compounds in simulations using the electromechanical model and drug‐ion channel data, compared to reported experimental and clinical data (as denoted in the key) Green triangles and orange squares show agreement and disagreement, respectively, whereas a circle denotes an unknown effect. Duloxetine and Paliperidone were the compounds selected for further investigations. *A*, simulation results using version 1 of the model; *B*, simulation results using version 2. [Correction made on 22 May, 2026 after first online publication: the preceding sentence has been updated to clearly specify that Duloxetine and Paliperidone were the compounds selected for further investigations.]

The experimental data used for comparison are mostly qualitative reports of inotropic effects without detailed calcium signalling or active tension measurements available. Figure 3*A* shows all the tested compounds classified by their effects on simulated active tension amplitude and includes a comparison with known experimental data.

Among 48 drugs with known clinical or experimental inotropic characteristics, the simulations using the baseline version 1 model accurately identified the effects of 38 drugs: 30 out of 35 with negative inotropic effects, 4 out of 7 with positive inotropic effects, and 4 out of 6 with no effects. This accuracy rate demonstrates that simulations with the electromechanical model and drug‐ion channel data are particularly robust in predicting the drug effects on contractility when considering negative inotropic drugs. For positive inotropy classification, additional inputs beyond IC50 values and Hill coefficients might be required to achieve higher accuracy in simulations.

Among the 48 drugs, simulations with version 2 of the model correctly predicted the effects of 41 compounds (Fig. 3*B*). Negative inotropic effects were particularly well captured, probably due to the upscaling of *I*_CaL_ in the model, which amplifies the impact of calcium channel blockade. Drugs with reported negative effect on inotropy (e.g. dysopirimidine, cisapride) were correctly indicated as negative inotropes by version 2 of the model, but not by version 1. Conversely, compounds with reported positive inotropy (e.g. cilostazol, BaCl_2_) were correctly identified as positive inotropes by version 1 and but version 2 of the model. Overall, version 2 of the model accurately identified the effects of 34 out of 35 negative inotropes and 1 out of 7 positive inotropes.

### Experimental validation of simulated contractility effects

After validating the model using drugs with known inotropic effects, we applied it to predict the potential inotropic effects of drugs that had not yet been experimentally tested in hiPSC‐CMs. Simulations of the activity of 16 drugs of unknown inotropic effect predicted several of these compounds to have positive or negative inotropic effects (Fig. 3). Changes in active tension observed in the simulations reflect underlying changes in calcium transient amplitude, as calcium transients are key drivers of contraction in cardiomyocytes.

Two drugs, duloxetine and paliperidone (Fig. 3), were selected to validate simulation predictions. Duloxetine and paliperidone were chosen as representative compounds of multichannel action and single‐channel action, respectively, both with unknown inotropic effects. Duloxetine, a serotonin–norepinephrine reuptake inhibitor, was simulated as a multichannel inhibitor (*I*_CaL_, *I*_Na_, *I*_Kr_) at 100‐fold effective free therapeutic plasma concentration (EFTPCmax), and as an *I*_CaL_ and *I*_Kr_ inhibitor at 10‐fold EFTPCmax, based on literature values (Passini et al., 2017). Paliperidone, an anti‐psychotic drug, was simulated as a single *I*_Kr_ channel blocker at its EFTPCmax and at 10‐fold EFTPCmax.

**Figure 4:**
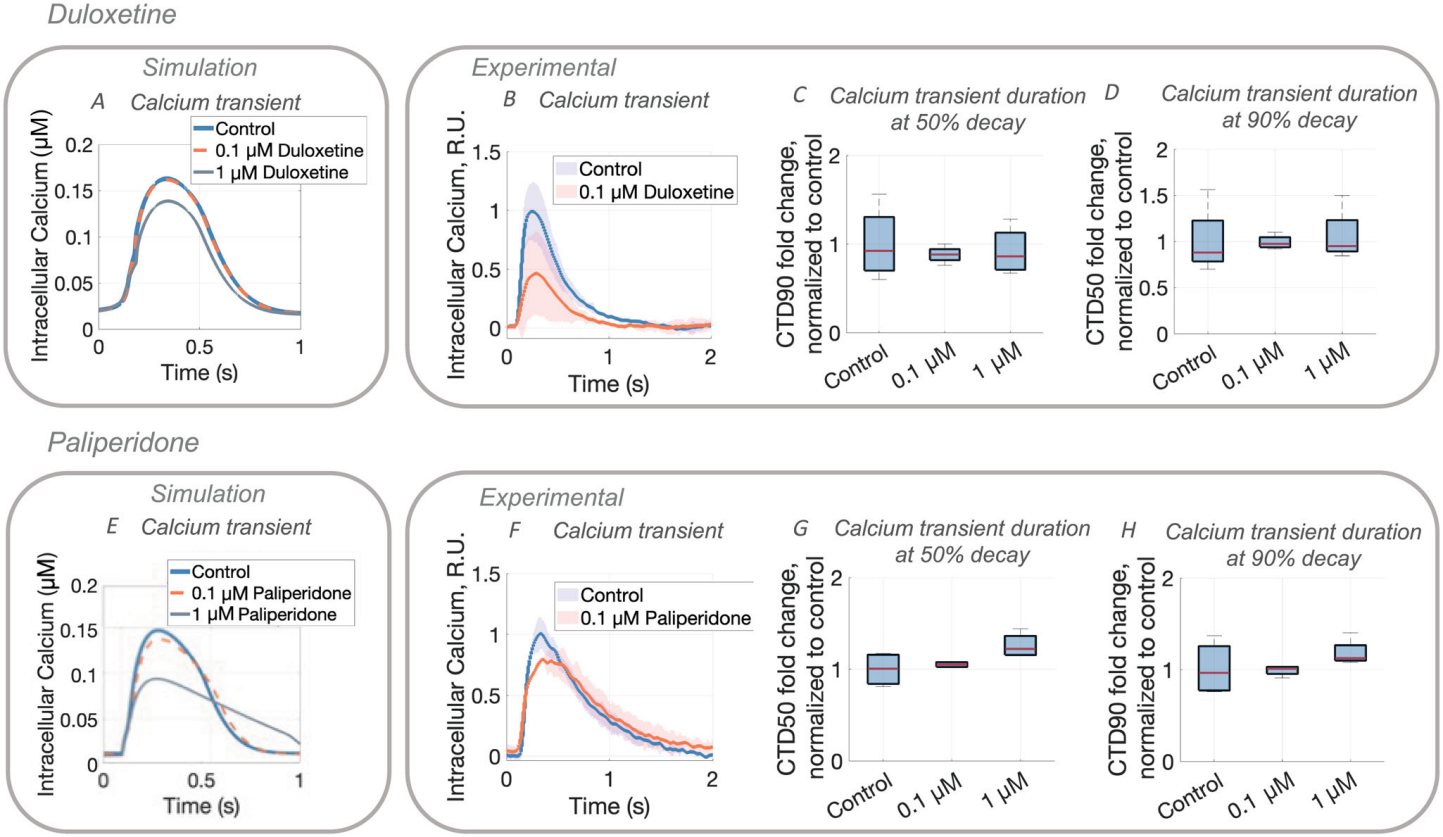
Comparison of experimental and simulation calcium data Shaded areas and error bars represent the SD. Data from at least three independent experiments. *A*, simulated hiPSC‐CM calcium transient following the application of duloxetine. *B*, average experimental hiPSC‐CM calcium transient following the application of duloxetine. *C*, experimental hiPSC‐CM data showing CTD50 following duloxetine application. *D*, experimental hiPSC‐CM data showing CTD90 following duloxetine application. *E*, simulated hiPSC‐CM calcium transient following the application of paliperidone. *F*, average experimental hiPSC‐CM calcium transient following the application of paliperidone. *G*, experimental hiPSC‐CM data showing CTD50 following paliperidone application. *H*, experimental hiPSC‐CM data showing CTD90 following paliperidone application.

**Figure 5:**
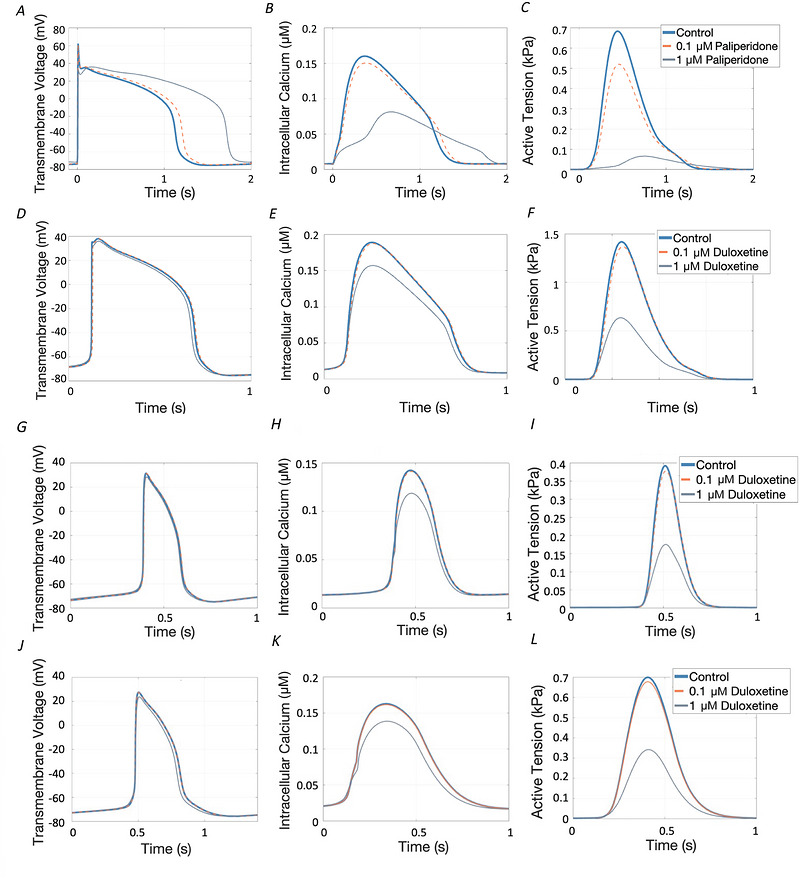
Simulations predict duloxetine and paliperidone effects on hiPSC‐CM action potentials, calcium transients and active tension *In silico* electromechanical hiPSC‐CM model simulations in paced mode following increasing doses of peliperidone show a change in: *A*, action potentials; B, calcium transients; and C, active tension at 20°C in paced cells (0.5 Hz). *In silico* electromechanical hiPSC‐CM model simulations in paced mode following increasing doses of duloxetine show a change in D, action potentials; E, calcium transients; and F, active tension at 20°C in paced cells (0.5 Hz). *In silico* electromechanical hiPSC‐CM model simulations at 37°C in paced mode following increasing doses of duloxetine show a change in G, action potentials; H, calcium transients; and I, active tension. *In silico* electromechanical hiPSC‐CM model simulations at 37°C in spontaneously beating cells following increasing doses of duloxetine show a change in *J*, action potentials; *K*, calcium transients; and *L*, active tension.

Simulations of paced hiPSC‐CMs show that 0.1 and 1 µM duloxetine have a negative inotropic effect (EFTPCmax = 0.016 µM, Fig. 4*A*). This effect is linked to duloxetine‐induced reductions in calcium transient amplitude and duration, as predicted by the model. To validate the predictions, the effects of duloxetine were experimentally tested in hiPSC‐CMs measuring the change in calcium transients. Simulations show that duloxetine induces a small reduction in calcium transient duration, which was subsequently observed experimentally when measuring 50% and 90% of calcium transient duration (CTD50 and CTD90) (Fig. 4*B*–*D*). Furthermore, both simulations and experimental data indicate a reduction in the calcium transient peak after the application of duloxetine, demonstrating a qualitative agreement between experimental observations and simulation results. In addition to predicting calcium transient markers, the hiPSC‐CM electromechanical model also predicted a shortening of action potential duration and a decrease in force (Fig. 5).

Simulations of 0.1 and 1 µM paliperidone (EFTPCmax = 0.069 µM) in paced hiPSC‐CMs suggest it has a negative effect on inotropy and prolongs the calcium transient duration (Fig. 4*E*). To further validate these predictions, the effects on calcium transients were tested experimentally in paced cells. The experimental calcium traces in paced cells also showed a prolonged calcium transient duration following paliperidone application in a dose‐dependent manner, consistent with *in silico* results (Fig. 4*F*–*H*).

Simulations with version 2 of the model showed results consistent with those reported in Fig. 4 and were in qualitative agreement with the waveforms illustrated in Figs 4 and 5. Action potential, calcium transient and active tension waveforms were similar between the two versions, indicating that the predictive behaviour for duloxetine and paliperidone in these settings was preserved.

These results establish the hiPSC‐CM electromechanical models as a robust platform for simulating drug‐induced inotropic effects with good agreement between experimental and simulation results. Mechanistically, the observed negative inotropic effects of duloxetine and paliperidone can be attributed to their respective actions on ion channels: duloxetine's inhibition of *I*_CaL_ (and other channels) and paliperidone's selective *I*_Kr_ blockade. Compared to simpler models, this approach captures the complex interactions between ion channel modulation and contraction control, providing a more comprehensive and mechanistic understanding of drug effects. Furthermore, unlike machine learning models, this framework can not only simulate novel drug effects, but also offer an interpretable tool for drug testing.

### Inotropic drug effects are affected by the hiPSC‐CM cell pacing mode

**Figure 6:**
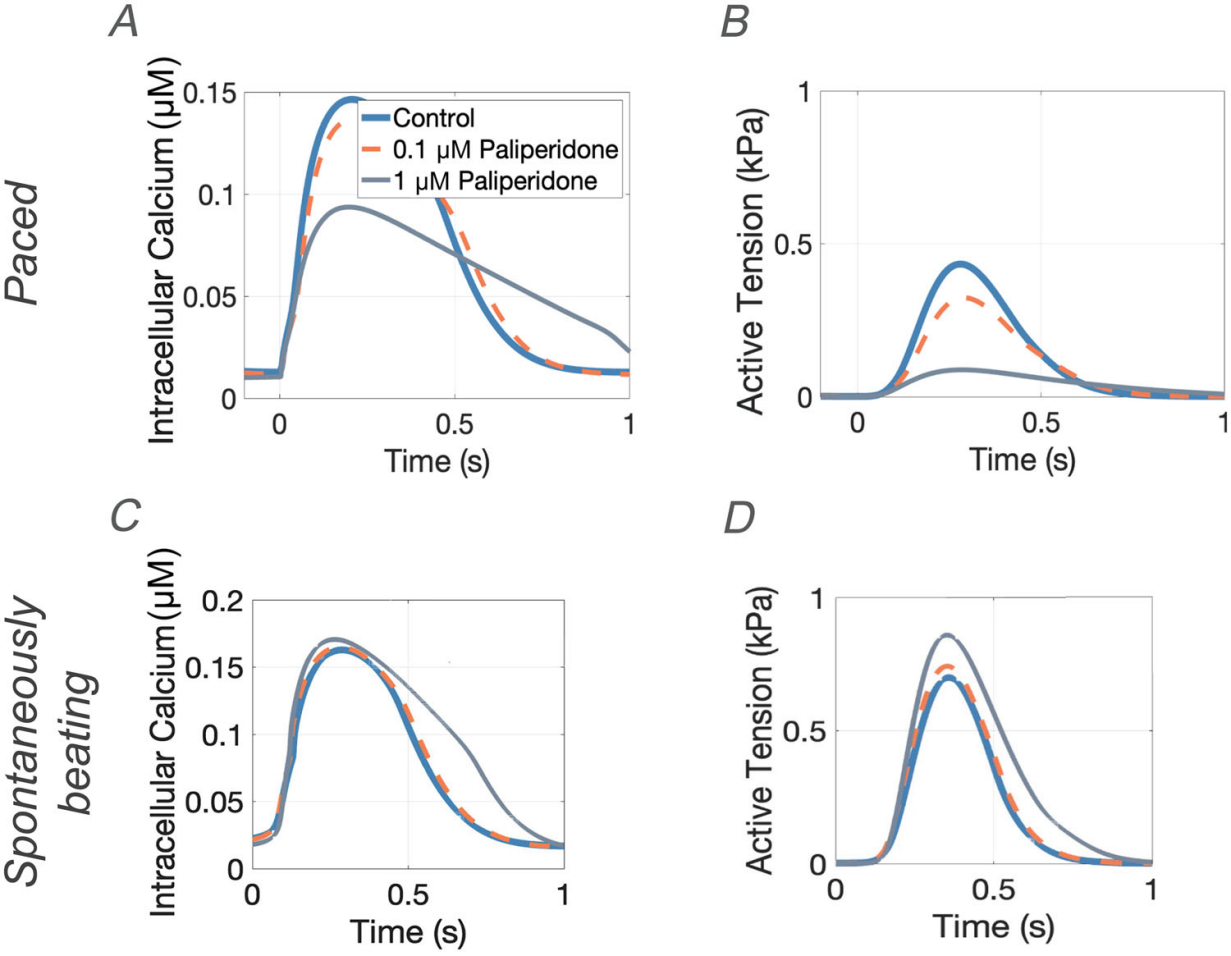
Paliperidone effects on active tension and calcium transient are dependent on pacing *A*, simulated calcium transient after paliperidone application in paced cells. *B*, simulated active tension after paliperidone application in paced cells. *C*, simulated calcium transient after paliperidone application in spontaneously beating cells. *D*, simulated active tension after paliperidone application in spontaneously beating cells.

Similar to the paced mode in Fig. 4, when simulated in a spontaneously beating hiPSC‐CM cell mode, duloxetine has a negative inotropic effect (Fig. 5). Simulations with version 1 of the model showed that unlike for duloxetine, the effects of paliperidone were different between the paced and the non‐paced modes. Although the calcium transient durations were prolonged in both modes following paliperidone application (Fig. 6*A*, *C*), the paced mode predicted a reduction in the peak force and calcium transient (Fig. 6*A*, *B*), whereas the non‐paced mode showed a mild increase of contractility (Fig. 6*C*, *D*).

We therefore used the model to explore what gives rise to the differences in calcium signalling and inotropy between paced and non‐paced cells. It was hypothesized that these differences might arise from the interplay between pacing mode and drug‐induced changes in *I*_CaL_. In paced cells, the shorter cycle length could result in reduced *I*_CaL_ availability due to insufficient recovery time for calcium channels between beats, leading to weaker *I*_CaL_ current and diminished inotropy. In contrast, in non‐paced cells, the longer cycle length may allow more time for *I*_CaL_ channels to recover, increasing calcium entry and resulting in higher calcium transient peaks and prolonged calcium transient duration. Alternatively, the differences observed might result from upstroke variations between the two pacing modes, or a more significant action potential prolongation induced by *I*_Kr_ blockade allowing for increased calcium entry during the plateau phase. To test these hypotheses, the cycle length in paced *versus* non‐paced cells was compared. Non‐paced cells have a prolonged cycle length, and the cycle length increases with increasing doses of paliperidone (paced: 1000 ms *vs*. 1856 ms in non‐paced at 1 µM paliperidone).

**Figure 7:**
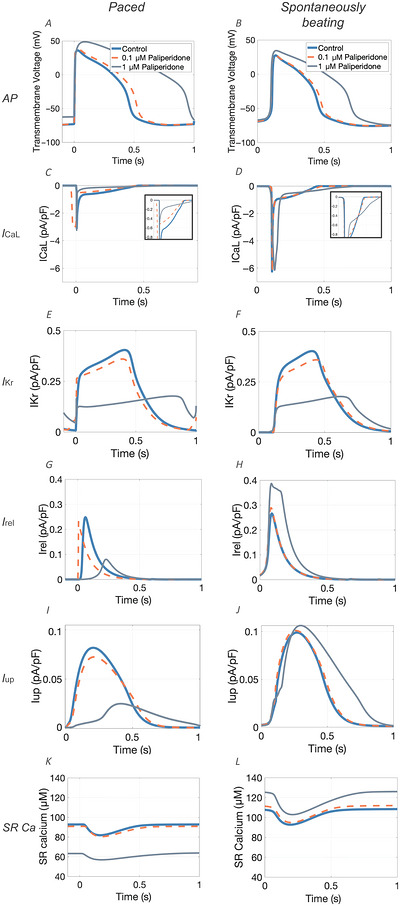
Paliperidone‐induced changes are dependent on the cell beating rate Paliperidone‐induced action potential changes in the paced (*A*) and the spontaneously beating (*B*) cell model. *I*_CaL_ current changes (*C*, *D*), *I*_Kr_ current changes (*E*, *F*), *I*_rel_ current changes (*G*, *H*), *I*_up_ current changes (*I*, *J*) and SR calcium loading changes (*K*, *L*) following paliperidone application in the paced and the spontaneously beating cell model.

Further comparisons of the action potential prolongations as well as the different currents between paced and non‐paced cells were then performed. Action potentials were prolonged to a greater degree in the paced than the spontaneously beating mode (Fig. 7*A*, *B*). Therefore, it was concluded that the increased calcium amplitude in the spontaneous beating mode is not due to a more significant action potential prolongation induced by *I*_Kr_ blockade. Generally, non‐paced cells had a greater *I*_CaL_ peak than paced cells. Since the longer plateau phase does not appear to correspond to a larger *I*_CaL_ in the paced cells (Fig. 7*A–D*), the larger *I*_CaL_ amplitude in Fig. 7*D* is therefore probably driven by the greater *I*_CaL_ availability. Rate‐dependent difference of paliperidone effects were also observed with version 2 of the model.

*I*_Kr_ currents were smaller and longer when affected by the drug, and these differences were driven by *I*_Kr_ current blockage (Fig. 7*E*, *F*). Changes in *I*_Kr_ prolong the repolarization phase and potentially delay the time until the pacemaker current can depolarize the cell to threshold again for the non‐paced mode. This lengthening cycle leads to a more prolonged diastolic interval in the non‐paced cells, allowing more time for the calcium handling system to recover, leading to increased *I*_CaL_ channel availability and a greater *I*_CaL_ peak, as well as a prolonged calcium reuptake into the sarcoplasmic reticulum in the non‐paced mode.

Consistent with this hypothesis, paliperidone induces an increased reuptake in non‐paced cells and reduced reuptake in paced cells (Fig. 7*I*, *J*). Significantly reduced *I*_CaL_ current in paced cells results in a lower amount of calcium being taken back to the SR and a reduced subsequent release in paced cells. This is unlike in spontaneously beating cells where greater *I*_CaL_ availability results in a greater amount of cytosolic calcium that can be taken back to the SR leading to a greater subsequent release. The calcium content in the SR is higher in the non‐paced mode than in the paced mode (Fig. 7*K*, *L*), which could be due to both higher *I*_CaL_ amplitude and longer *I*_up_. The elevated calcium loading state in the SR of the non‐paced cell model subsequently leads to a higher amplitude of calcium release (*I*_rel_) (Fig. 7*G*, *H*). This mechanism can explain why calcium transient peaks are not reduced but are slightly greater in non‐paced cells, as more calcium available in the SR can lead to more substantial releases during each excitation.

**Figure 8:**
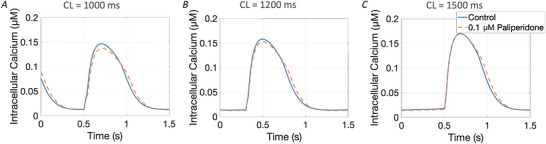
The effects of paliperidone are beating rate‐dependent *A*, paliperidone induces a reduction in calcium transient peak in the cell model paced at 1 Hz. *B*, paliperidone induces a reduction in calcium transient peak in cell paced at 50 beats per minute. The reduction in calcium transient amplitude is less pronounced than in cell paced at 60 beats per minute. *C*, paliperidone does not induce a reduction in calcium transient peak in the cell model paced at 40 beats per minute unlike in cells paced at 60 beats per minute (*A*). CL, cycle length.

To verify the above‐mentioned mechanisms, we tested whether slower pacing with paliperidone would lead to similar inotropic effects as observed in the spontaneous mode. Upon prolonging the cycle length, it was observed that the negative inotropic effects of paliperidone were no longer present (Fig. 8).

In summary, the simulations show that the differences in paliperidone‐induced inotropic effects between the spontaneously beating and paced cells arise from their differences in *I*_CaL_ availability. Altogether, this demonstrates that the model enables mechanistic investigations of drug‐induced signalling. In this case, simulations demonstrate that the drug effects on calcium dynamics and contractility are dependent on the beating rate.

## Discussion

This study describes the development, calibration and experimental evaluation of two versions of electromechanical hiPSC‐CM models for simulations of active tension, calcium transient and cellular electrophysiology including the action potential and ionic currents. Version 2 was developed from Version 1 by incorporating experimentally observed increases in *I*_Na_, *I*_CaL_ and *I*_K1_ currents measured in hiPSC‐CMs following long‐term culture. As a result, Version 2 exhibited calcium transients and active tension dynamics that were more similar to those of adult cardiomyocytes. Collectively, the two model versions captured the heterogeneity observed in experimental hiPSC‐CM data.

The key findings described in this study are:

1. Simulations with the hiPSC‐CM electromechanical model and standard drug‐ion channel data yield good qualitative agreement with inotropic effects reported in experimental data for 48 compounds. Version 1 of the model correctly identified the inotropic activity of 38 drugs, and version 2 that of 41 drugs.
2. The simulations identify previously unknown drug effects, which were later confirmed experimentally for two selected drugs.
3. Mechanistic insights from the model reveal that paliperidone‐induced effects are linked to calcium handling processes, with key differences observed between paced and non‐paced cells.

By integrating *in silico* results with *in vitro* data, the models provide a mechanistic understanding of hiPSC‐CM behaviour under pharmacological interventions together with a framework that can be continuously refined through iterative simulations and experimental data. The integrated approach enables novel insights into the mechanisms driving drug‐induced inotropic effects, thus advancing beyond the limitations of standard drug screening models.

Standard drug‐ion channel data consisting of IC50 values and Hill coefficients were used as input to the models (Passini et al., 2017). This is to facilitate translation to a wide variety of drug development platforms and workflows. We demonstrate that simulations successfully predicted the drug responses of 41 compounds. This highlights the model's effectiveness in simulating drug responses despite minimal input, addressing a critical challenge in pharmacological modelling. Our positive results align with comparable simulations in human adult cardiomyocyte models (Passini et al., 2017), and build upon previously established electrophysiology‐only hiPSC‐CM models evaluated with action potential measurements (Paci et al., 2020).

For integration of the hybrid *in vitro–in silico* system, simulations and new experiments were conducted for compounds with unknown effects on contractility and calcium: duloxetine and paliperidone. The simulation/experiment agreement illustrates their use to fill critical knowledge gaps in drug–cardiomyocyte interactions, offering a powerful tool for early‐phase drug safety assessment.

The baseline hiPSC‐CM electromechanical model was used to simulate rate‐dependent effects, which is critical for accurate drug safety assessments. The study demonstrates that paliperidone has distinct effects in paced and spontaneously beating cells. Specifically, it was shown that the decrease in inotropic response in paced cells, which was not observed in spontaneously beating cells, probably results from paliperidone‐induced effects on L‐type calcium current. Our analysis supports the model in which the increased *I*_CaL_ availability results in greater calcium content in the cell. This could lead to increased inotropy and a higher calcium transient peak in spontaneously beating cells, but not in paced cells. This rate‐dependent behaviour highlights the importance of considering pacing conditions when evaluating drug effects on contractile function.

Currently, the contractile effects of duloxetine and paliperidone in human cardiac cells are unknown. Testing these effects and understanding the underlying mechanisms is important, especially given the wide prescription of duloxetine, one of the first‐line antidepressants used globally, and its designation as a drug against hot flushes (Biglia et al., 2018) and menopause‐related symptoms (Joffe et al., 2007). This is the first time the inotropic effects of duloxetine and paliperidone have been established *in silico* in hiPSC‐CMs and validated with a novel set of calcium handling experimental data. Understanding these drugs’ inotropic effects could lead to safer prescribing practices and potentially guide the development of new therapeutic strategies.

iPSC‐CM electromechanical models enable a more comprehensive assessment of drug effects than purely electrophysiological models (Paci et al., 2020). Additionally, unlike previous studies with adult cardiomyocytes, models enable hiPSC‐CM‐specific simulations, which are increasingly relevant in personalized medicine and early drug testing (Passini et al., 2017). Studies by Li et al. (2020) and Ovics et al. (2020) focus on hiPSC‐CMs for drug screening, highlighting the relevance of this work. Compared to these studies, the presented *in silico* approach together with the integration of mechanical responses offers an advancement in understanding drug–cardiomyocyte interactions, addressing a critical gap in current hiPSC‐CM research. Furthermore, the model framework extends the capabilities of testing drug activity in the wet lab by offering simulations of inotropy, action potential and ionic currents in a quick, accurate and user‐friendly manner. Achieving similar results through wet‐lab methods alone would require significantly more time, labour and cost.

The new baseline model is calibrated and validated on hiPSC‐CM and human data. This represents an advancement relative to a previously published electro‐mechanical hiPSC‐CM model (Forouzandehmehr et al., 2021), which integrates the Rice et al. (2008) myofilament model together with the hiPSC‐CM electrophysiology model developed by Paci et al. (2020). The Rice model was designed to reproduce a wide range of myofilament behaviours, including steady‐state and twitch force–calcium relations, force–velocity relationships and sarcomere length dependence, and has been widely used in cardiac modelling studies. However, it was originally calibrated using data obtained from rodent (rat) and rabbit ventricular myocytes, primarily at sub‐physiological temperatures (22–30°C). Therefore, its direct application to human or hiPSC‐CM simulations may not fully capture human‐specific contractile behaviour, particularly in the context of hiPSC‐CM modelling at 37°C, as assumed in the Paci model.

In contrast, the Land model was explicitly developed using experimental measurements from skinned human cardiomyocytes at body temperature (37°C). It incorporates human‐specific kinetics of troponin C binding, thin filament activation and cross‐bridge cycling. Additionally, the model includes viscoelastic and length‐dependent components of contraction derived from human data. When integrated into whole‐heart finite element simulations, the Land model accurately reproduces human pressure–volume loops and ejection fractions, demonstrating its suitability for translational applications (Wang et al., 2021; Zhou et al., 2024).

Thus, replacing the Rice model with the Land model in the current framework improves physiological consistency by aligning both the electrophysiological and mechanical components with human data, enhancing the representation of human iPSC‐CM contractile function. [Correction made on 22 May 2026, after first online publication: The preceding three paragraphs have been updated to clarify that the electromechanical hiPSC‐CM model of Forouzandehmehr et al. (2021) relies on an established contractile formulation that is adapted using hiPSC‐derived measurements.]

Finally, the baseline model's capabilities reinforce the value of integrating electromechanical aspects into hiPSC‐CM drug testing frameworks. This streamlined process supports the broader application of the framework in drug discovery and safety pharmacology.

### Limitations

The framework provides a new important approach for mechanistic signalling investigations in cardiomyocytes, but it is not without limitations. The model simulations show limited accuracy for positive inotrope substances, especially version 2 of the model. Further refinement is necessary to enhance the accuracy of simulating responses for positive inotropes, which will depend on acquiring more detailed experimental data on drug‐specific mechanisms as well as data on contraction kinetics. Additionally, a limitation arises from the challenges in assessing mechanics in hiPSC‐CMs, as their sarcomeres are not well organized and forces are usually measured radially, as done in this study. This affects the reliability of force measurements.

Our study also highlights the importance of pacing in modulating drug effects, as shown by several experimental and clinical reports. Whereas our baseline model includes rate‐dependent properties, standard drug‐ion channel data do not. These could be integrated if deemed appropriate for specific studies such as in the case of flecainide (Wang et al., 1993; Yang et al., 2021).

The calcium handling in the current model is inherited from Paci et al. (2020), which is based on data with lower absolute [Ca^2^^+^] values than more recent experimental reports. In version 2 of the model, we have updated the calcium homeostasis to reflect newer findings (e.g. Seibertz et al., 2023) to provide a more accurate representation of the physiological calcium signalling in hiPSC‐CMs. In addition, TRPN and CMDN concentrations in the models were scaled from an adult cardiomyocyte model due to the lack of quantitative data specific to hiPSC‐CMs. While functionally consistent calcium transients were achieved, in the future it would be useful to test different approaches in modelling calcium buffering protein expression differences between fetal‐like hiPSC‐CMs and adult cells.

The baseline model was calibrated using heterogeneous experimental datasets (e.g. data acquired in different temperatures), and inter‐experimental variability was not explicitly accounted for. While this reduces the risk of classical overfitting, it may limit interpretability and generalizability to specific experimental conditions. Future work could incorporate structured variability modelling to address this. Also, some of the experiments for comparing drug response to simulated datasets were performed using a single hiPSC‐CM dataset at 20°C. Broader validation across multiple hiPSC‐CM lines and different temperatures would be useful for improving the validation accuracy in the future. Also, drug response validation was performed against heterogeneous experimental and clinical datasets generated under varying conditions. Direct comparison for selected compounds at matched experimental conditions would strengthen confidence in the predictive performance in the future.

Future work could also include modelling of additional signalling pathways that play an important role in mediating cellular contraction as well as the inclusion of more sophisticated drug‐binding kinetics beyond Hill coefficients and IC50 values, and detailed pharmacodynamic models to better predict the temporal aspects of drug action. Additionally, expanding the baseline model to incorporate additional mechanical–electrical feedback mechanisms between electrical activity and mechanical responses could enhance the simulation of complex drug effects and disease. By incorporating these refinements, hiPSC‐CM electromechanical models could achieve greater accuracy and reliability in predicting cardiomyocyte behaviour under various pharmacological interventions.

## Conclusions

We describe the development, calibration and validation of new hiPS‐CM electromechanical models for the simultaneous simulation of drug effects on active tension, calcium transient and cellular electrophysiology. These tools serve as bridges between theoretical simulations and practical applications in cardiac safety and efficacy studies.

## Additional information

## Competing interests

The authors confirm no competing interests.

## Author contributions

Conception and design of the work: B.R., M.Z., X.Z., M.F. Acquisition, analysis and interpretation of data for the work: M.F., X.Z., A.K., Y.H., M.C., M.K., U.R., R.P. Drafting the work or revising it critically for important intellectual content: M.F., X.Z., A.K., Y.H., M.C., M.K., U.R., R.P., M.Z., B.R. Final approval of the version to be published: M.F., X.Z., A.K., Y.H., M.C., M.K., U.R., R.P., M.Z., B.R. Agreement to be accountable for all aspects of the work: M.F., X.Z., A.K., Y.H., M.C., M.K., U.R., R.P., M.Z., B.R.

## Funding

MF (BHF, FS/4yPhD/F/20/34132); UR, MC, RP (Marie Skłodowska‐Curie grant no. 860974, Collaborative Research Centre CRC1425 (DFG no. 422681845)); XZ (Oxford‐Bristol Myers Squibb Fellowship, R39207/CN063); MZ (BHF, PG/15/5/31110, RG/17/6/32944); BR (Wellcome Trust, 214290/Z/18/Z; NC3Rs, NC/P001076/1).

## Supporting information


Peer Review History

## Data Availability

All data generated or analysed during this study are included in this article. All source code used in this paper is available and can be obtained by emailing corresponding authors.
