# Supplementary material for: Electromechanical modelling and simulation of human‐induced pluripotent stem cell‐derived cardiomyocytes predict drug‐induced contractility effects
Source: J Physiol. 2026 Mar 11;604(13):5629–62. doi: 10.1113/JP288623 (PMC13327774; doi:10.1113/JP288623)
Supplement: Supplementary file 1 — Peer Review History [file TJP-604-5629-s001.pdf]

The Journal of Physiology

Electromechanical Modelling and Simulation of hiPSC-Derived Cardiomyocytes Predict Drug Induced Contractility Effects

Milda Folkmanaite, Xin Zhou, Andreas Koschinski, Wan-Hua Hong, Marilù Casini, Manuel Koch, Rémi Peyronnet, Ursula Ravens, Manuela Zaccolo, and Blanca Rodriguez
DOI: 10.1113/JP288623

Corresponding author(s): Milda Folkmanaite (mildafolkmanaite@gmail.com)

The following individual(s) involved in review of this submission have agreed to reveal their identity: Jussi Koivumäki (Referee #1); Claudia Crocini (Referee #2)

Review Timeline:

Submission Date:	30-Jan-2025
Editorial Decision:	04-Mar-2025
Revision Received:	26-Apr-2025
Editorial Decision:	15-May-2025
Revision Received:	16-Jan-2026
Accepted:	02-Feb-2026

Senior Editor: Bjorn Knollmann

Reviewing Editor: Eleonora Grandi

Transaction Report:

(Note: With the exception of the correction of typographical or spelling errors that could be a source of ambiguity, letters and reports are not edited. Depending on transfer agreements, referee reports obtained elsewhere may or may not be included in this compilation. Referee reports are anonymous unless the Referee chooses to sign their reports.)

Dear Dr Folkmanaite,

Re: JP-RP-2025-288623 "**Electromechanical Modelling and Simulation of hiPSC-Derived Cardiomyocytes Predict Drug Induced Contractility Effects**" by Milda Folkmanaite, Xin Zhou, Andreas Koschinski, Wan-Hua Hong, Marilu Casini, Manuel Koch, Rémi Peyronnet, Ursula Ravens, Manuela Zaccolo, and Blanca Rodriguez

Thank you for submitting your manuscript to The Journal of Physiology. It has been assessed by a Reviewing Editor and by 2 expert referees and we are pleased to tell you that it is potentially acceptable for publication following satisfactory major revision.

Please advise your co-authors of this decision as soon as possible.

The referee reports are copied at the end of this email.

Please address all the points raised and incorporate all requested revisions or explain in your Response to Referees why a change has not been made. We hope you will find the comments helpful and that you will be able to return your revised manuscript within 2 months. If you require longer than this, please contact journal staff: jp@physoc.org. Please note that this letter does not constitute a guarantee for acceptance of your revised manuscript.

Your revised manuscript should be submitted online using the link in your Author Tasks: Link Not Available. This link is accessible via your account as Corresponding Author; it is not available to your co-authors. If this presents a problem, please contact journal staff (jp@physoc.org). Image files from the previous version are retained on the system. Please ensure you replace or remove any files that are being revised.

If you do not wish to submit a revised version of your manuscript, you must inform our journal staff (jp@physoc.org) or reply to this email to request withdrawal. Please note that a manuscript must be formally withdrawn from the peer review process at one journal before it may be submitted to another journal.

TRANSPARENT PEER REVIEW POLICY: To improve the transparency of its peer review process, The Journal of Physiology publishes online as supporting information the peer review history of all articles accepted for publication. Readers will have access to decision letters, including Editors' comments and referee reports, for each version of the manuscript, as well as any author responses to peer review comments. Referees can decide whether or not they wish to be named on the peer review history document.

ABSTRACT FIGURES: Authors are expected to use The Journal's premium BioRender account to create/redraw their Abstract Figures. Information on how to access this account is here: https://physoc.onlinelibrary.wiley.com/journal/14697793/biorender-access.

This will enable Authors to create and download high-resolution figures. If authors have used the free BioRender service, they can use the instructions provided in the link above to download a high-resolution version suitable for publication.

The link provided should only be used for the purposes of this submission. Authors will be charged for figures created on this account if they are not related to this manuscript submission.

LANGUAGE EDITING AND SUPPORT FOR PUBLICATION: If you would like help with English language editing, or other article preparation support, Wiley Editing Services offers expert help, including English Language Editing, as well as translation, manuscript formatting, and figure formatting at www.wileyauthors.com/eeo/preparation. You can also find resources for Preparing Your Article for general guidance about writing and preparing your manuscript at www.wileyauthors.com/eeo/prepresources.

RIGOUR AND REPRODUCIBILITY: We recommend authors consult The Journal's Rigour and Reproducibility requirements to ensure that their manuscript is suitable for publication: https://physoc.onlinelibrary.wiley.com/pb-assets/hub-assets/physoc/documents/TJP-Rigour-and-Reproducibility-Requirements.pdf

REVISION CHECKLIST:

Check that your Methods section conforms to journal policy: https://jp.msubmit.net/cgi-bin/main.plex?form_type=display_requirements#methods.

Check that data presented conforms to the statistics policy: https://jp.msubmit.net/cgi-bin/main.plex?form_type=display_requirements#statistics.

Upload a full Response to Referees file. To create your 'Response to Referees': copy all the reports, including any comments from the Senior and Reviewing Editors, into a Microsoft Word, or similar, file and respond to each point, using font or background colour to distinguish comments and responses and upload as the required file type.

Please upload two versions of your manuscript text: one with all relevant changes highlighted and one clean version with no changes tracked. The manuscript file should include all tables and figure legends, but each figure/graph should be uploaded as separate, high-resolution files.

You may also upload:

- 'Potential Cover Art' for consideration as the issue's cover image
- Appropriate Supporting Information (Video, audio or data set: see https://jp.msubmit.net/cgi-bin/main.plex?form_type=display_requirements#supp).

We look forward to receiving your revised submission.

If you have any queries, please reply to this email and we will be pleased to advise.

Yours sincerely,

Bjorn Knollmann
Senior Editor
The Journal of Physiology

REQUIRED ITEMS

- Include a Key Points list in the article itself, before the Abstract.

- Author photo and profile. First or joint first authors are asked to provide a short biography (no more than 100 words for one author or 150 words in total for joint first authors) and a portrait photograph. These should be uploaded and clearly labelled together in a Word document with the revised version of the manuscript. See Information for Authors for further details.

- The reference list must be in alphabetical order, rather than numbered, to comply with our Journal format.

- Your manuscript must include a complete Additional Information section, including competing interests; funding; author contributions and acknowledgements.

- Please upload separate high-quality figure files via the submission form.

- Please ensure that any tables are editable and in Word format, and wherever possible, embedded in the article file itself.

- Your paper contains Supporting Information of a type that we no longer publish, including supplementary tables and figures. Any information essential to an understanding of the paper must be included as part of the main manuscript and figures. The only Supporting Information that we publish are video and audio, 3D structures, program codes and large data files. Your revised paper will be returned to you if it does not adhere to our Supporting Information Guidelines.

- Please include an Abstract Figure file, as well as the Figure Legend text within the main article file. The Abstract Figure is a piece of artwork designed to give readers an immediate understanding of the research and should summarise the main conclusions. If possible, the image should be easily 'readable' from left to right or top to bottom. It should show the physiological relevance of the manuscript so readers can assess the importance and content of its findings. Abstract Figures should not merely recapitulate other figures in the manuscript. Please try to keep the diagram as simple as possible and without superfluous information that may distract from the main conclusion(s). Abstract Figures must be provided by authors no later than the revised manuscript stage and should be uploaded as a separate file during online submission labelled as File Type 'Abstract Figure'. Please also ensure that you include the figure legend in the main article file. All Abstract Figures

should be created using BioRender. Authors should use The Journal's premium BioRender account to export high-resolution images. Details on how to use and access the premium account are included as part of this email.

EDITOR COMMENTS

Reviewing Editor:

Both reviewers commented favorably on the manuscript, but found several areas for improvement. These mostly involve improved clarity (e.g., figure quality and font sizes, details of experimental protocols) and attention to aspects of model calibration and validations. Please carefully revise the manuscript by addressing all reviewers' comments.

Please also see 'Required Items' above.

Senior Editor:

I concur with the reviewing editor's assessment and recommendations.

REFEREE COMMENTS

Referee #1:

Folkmanaite et al. represent an electromechanical in silico hiPSC-CM model that couples the Paci et al. (2020) electrophysiology and Land et al. (2017) biomechanical models. The authors simulated the effects of 63 different drugs, including ones with previously untested inotropic effects, to demonstrate the utility of the developed model. Furthermore, Folkmanaite et al. elucidate the rate-dependent inotropic effects of paliperidone.

I have four (4) major concerns to be addressed and modified accordingly:

1) About the novelty of the model. The authors state on lines 89-91 that "The model is based on hiPSC-CM and human data only, making it the first of its kind, as previous electromechanical hiPSC-CM models have included different contractile elements from various species (10)."

This statement has two unclear points. Firstly, what are the "models" (in plural) that the authors are referring to, as there is only one reference? Secondly, if there is only one such model (as there is just one reference), that model probably does not include different contractile elements? Overall, this statement is a rather rough overselling of the novelty of the authors own work and oversimplifying of the previous electromechanical modelling by Forouzandehmehr et al. (2021) that they are citing. In the Forouzandehmehr et al. paper, the Rice et al. (2008) contractile element model was integrated into the Paci et al. (2020) hiPSC-CM electrophysiology model. The contractile element models by Rice et al. (2008) and Land et al. (2017) have each their pros and cons. As the authors probably know, the Rice model has been previously adapted to different species beyond the original rat implementation. Thus, its adaptation to hiPSC-CM does not inherently lead some vague cross-species problems.

Instead of that vague selling argument - pardon my French - please provide some concrete points, why the Land contractile element model is better, more human, etc. than the Rice model.

2) About the credibility of the model. The authors state on lines 91-98: "The credibility of the model is based on the human data and knowledge integrated in the equations and parameters, and also the comparison of simulation results against experimental data, including: hiPSC-CM contractility (contraction and relaxation duration, peak amplitude), calcium signalling (calcium transient duration), action potentials (action potential duration) (10-12), and drug effects observed experimentally for 63 different compounds, including those with both known and unknown effects on hiPSC-CM behaviour."

Since the main novelty of the model is the calibrated contractility, it warrants a proper calibration of the main messenger for that function: intracellular calcium signalling. The electrophysiology part of the hiPSC-CM model (Paci et al. 2020) inherits its calcium signalling from the 2018-version of the Paci model (https://doi.org/10.3389/fphys.2018.00709). Since then, at least Kernik et al. (2019; https://doi.org/10.1113/JP277724) and Seibertz et al. (2023; https://doi.org/10.1007/s00395-022-00973-0) have shown that diastolic and systolic calcium concentrations in hiPSC-CMs have the same magnitude that is common to mammalian cardiomyocytes. That is, diastolic [Ca2+] is about 100-200 nM and systolic about 400-500 nM. The data by Rast et al. (2015; https://doi.org/10.1016/j.vascn.2015.04.005), on which the parameterisation of 2018-Paci model is based, appears to be an anomaly with its super low [Ca2+] values. One can only speculate that maybe there was some technical problem that Rast and colleagues were unaware of.

So, instead of full-on recalibrating the Land et al. (2017) contractile element model, reparameterise first the calcium handling of the Paci et al. (2020) electrophysiology model to match the in vitro data of Kernik et al. (2019) and Seibertz et al. (2023). Then, the calcium dynamics of the hiPSC-CM model will be so close to native human CMs that likely only a small-scale parameter fine-tuning is needed for the Land et al. (2017) contractile element integration.

3) About the figures. Many of the figures are either as a whole very difficult to read or contain subpanels that are close to impossible to read. Understandably, there might be some resolution issues, when submitting the figures. However, that does not appear to be the main issue with the figures of this manuscript. Some specific notes:

* Of the figures that include plotted traces (Figures 2, 5-8) only Figure 6 has panel sizes and text font sizes that support the readability of the figure.

* Please, try to make good use of the available space in the figure layouts to illustrate the data in as informative way as possible, instead of having so much white space between the panels. The worst example of not doing that is Figure 2, where the x- and y-axes weirdly truncated. The axes could be stretched at least 2-fold or maybe even 2.5-fold, to use the white space in more meaningful way.

* What is the point of repeating the same legend multiple times in one figure, if the legend is the same for all panels? This applies to Figure 6 and 7.

* Figure 2: Try to condense the legend texts, so that they do not take so much space in the figure. Figure 2D-F: Please add biomarker values for the calibrated model for CTTP, CTD50, APD50, and APD90 in a same way as in panels A and B. Figure 2E: Please add experimental range for APD50, to be coherent with panel F.

4) Please state clearly the compliance with the human and/or animal ethics policy of the Journal and provide the required information. Now, there is only a brief note on lines 266-267 that "...human induced pluripotent stem cells (hiPSCs; M398 line (19)) were cultured as described before(20, 21)." To my understanding that does not meet requirements of the Journal.

Minor points to consider and fix:

* I'll make these notes, if needed, to the revised manuscript.

Referee #2:

This study by Folkmanaite et al., presents a novel electromechanical model of hiPSC-CMs with promising applications in drug testing.

Some points:

- The derivation of TRPN and CMDN concentrations assumes proportionality from the adult ToR-ORd model (Tomek et al.,

2017), despite known differences in calcium handling between hiPSC-CMs (fetal-like) and adult cardiomyocytes. For example, hiPSC-CMs exhibit reduced SERCA2a activity and higher reliance on transsarcolemmal calcium flux compared to adult cells.

- The calibration uses pooled experimental data from heterogeneous sources (Mixed 2D monolayers and 3D engineered heart tissues), variable pacing rates (0.2-2 Hz) and different temperatures (RT vs. 37{degree sign}C). How did the authors account for inter-experimental variability. Would this approach risk overfitting to non-generalizable conditions?

- How were the weights assigned for the weighted sum for TP, RT50, etc.?

- I am quite confused by the MaxAT values during pacing. Experimental values are an order of magnitude different.

- I can't find details on the doses used in the simulation and/or experimental available data used for comparison

- It would be nice to see experimental validation also of 1 or 2 drugs that are in agreement and 1 or 2 that are not in agreement to clarify whether your experimental validation pipeline is comparable to the literature. What was the temperature used for the simulations of the 63 drugs? Were the experimental data (from the literature) generated at the same temperature?

- The validating experiments (and simulations) at 20C are bit underwhelming. Also, it is a very good practice to validate experiments on multiple hiPSC lines.

- In the methods: "Differentiation into cardiomyocytes was induced in a monolayer as described previously." no citation is provided. Also how old were the cells at the moment of experiments? Where the cells tested for purity? Any maturation strategy used?

END OF COMMENTS

Response to Referees

REQUIRED ITEMS

- Include a Key Points list in the article itself, before the Abstract.

Key points have been included in the manuscript (page 2).

- Author photo and profile. First or joint first authors are asked to provide a short biography (no more than 100 words for one author or 150 words in total for joint first authors) and a portrait photograph. These should be uploaded and clearly labelled together in a Word document with the revised version of the manuscript. See Information for Authors for further details.

The first author's photo and biography have been included.

- The reference list must be in alphabetical order, rather than numbered, to comply with our Journal format.

This has been addressed now.

- Your manuscript must include a complete Additional Information section, including competing interests; funding; author contributions and acknowledgements.

Competing interests, funding, author contributions and acknowledgement are included in the manuscript (page 41).

- Please upload separate high-quality figure files via the submission form.

The figures have been uploaded.

- Please ensure that any tables are editable and in Word format, and wherever possible, embedded in the article file itself.

The tables throughout the document are editable.

- Your paper contains Supporting Information of a type that we no longer publish, including supplementary tables and figures. Any information essential to an understanding of the paper must be included as part of the main manuscript and figures. The only Supporting Information that we publish are video and audio, 3D structures, program codes and large data files. Your revised paper will be returned to you if it does not adhere to our Supporting Information Guidelines.

The information from the Supporting Information has been moved to the main article except for one large data file.

- Please include an Abstract Figure file, as well as the Figure Legend text within the main article file. The Abstract Figure is a piece of artwork designed to give readers an immediate understanding of the research and should summarise the main conclusions. If possible, the image should be easily 'readable' from left to right or top to bottom. It should show the physiological relevance of the manuscript so readers can assess the importance and content of its findings. Abstract Figures should not merely recapitulate other figures in the manuscript. Please try to keep the diagram as simple as possible and without superfluous information that may distract from the main conclusion(s). Abstract Figures must be provided by authors no later than the revised manuscript stage and should be uploaded as a separate file during online submission labelled as File Type 'Abstract Figure'. Please also ensure that you include the figure legend in the main article file. All Abstract Figures should be created using BioRender. Authors should use The Journal's premium BioRender account to export high-resolution images. Details on how to use and access the premium account are included as part of this email.

The abstract figure has been included.

Referee #1.

1) About the novelty of the model. The authors state on lines 89-91 that "The model is based on hiPSC-CM and human data only, making it the first of its kind, as previous electromechanical hiPSC-CM models have included different contractile elements from various species (10)."

   This statement has two unclear points. Firstly, what are the "models" (in plural) that the authors are referring to, as there is only one reference? Secondly, if there is only one such model (as there is just one reference), that model probably does not include different contractile elements? Overall, this statement is a rather rough overselling of the novelty of the authors own work and oversimplifying of the previous electromechanical modelling by Forouzandehmehr et al. (2021) that they are citing. In the Forouzandehmehr et al. paper, the Rice et al. (2008) contractile element model was integrated into the Paci et al. (2020) hiPSC-CM electrophysiology model. The contractile element models by Rice et al. (2008) and Land et al. (2017) have each their pros and cons. As the authors probably know, the Rice model has been previously adapted to different species beyond the original rat implementation. Thus, its adaptation to hiPSC-CM does not inherently lead some vague cross-species problems.

   Instead of that vague selling argument - pardon my French - please provide some concrete points, why the Land contractile element model is better, more human, etc. than the Rice model.

The wording has been edited as per the referee’s suggestion. The text (lines 756 – 782, pages 34-35) has been expanded to include the differences between Rice and Land models.

2) About the credibility of the model. The authors state on lines 91-98: "The credibility of the model is based on the human data and knowledge integrated in the equations and parameters, and also the comparison of simulation results against experimental data, including: hiPSC-CM contractility (contraction and relaxation duration, peak amplitude), calcium signalling (calcium transient duration), action potentials (action potential duration) (10-12), and drug effects observed experimentally for 63 different compounds, including those with both known and unknown effects on hiPSC-CM behaviour."

   Since the main novelty of the model is the calibrated contractility, it warrants a proper calibration of the main messenger for that function: intracellular calcium signalling. The electrophysiology part of the hiPSC-CM model (Paci et al. 2020) inherits its calcium signalling from the 2018-version of the Paci model (https://doi.org/10.3389/fphys.2018.00709). Since then, at least Kernik et al. (2019; https://doi.org/10.1113/JP277724) and Seibertz et al. (2023; https://doi.org/10.1007/s00395-022-00973-0) have shown that diastolic and systolic calcium concentrations in hiPSC-CMs have the same magnitude that is common to mammalian cardiomyocytes. That is, diastolic [Ca2+] is about 100-200 nM and systolic about 400-500 nM. The data by Rast et al. (2015; https://doi.org/10.1016/j.vascn.2015.04.005), on which the parameterisation of 2018-Paci model is based, appears to be an anomaly with its super low [Ca2+] values. One can only speculate that maybe there was some technical problem that Rast and colleagues were unaware of.

   So, instead of full-on recalibrating the Land et al. (2017) contractile element model, reparameterise first the calcium handling of the Paci et al. (2020) electrophysiology model to match the in vitro data of Kernik et al. (2019) and Seibertz et al. (2023). Then, the calcium dynamics of the hiPSC-CM model will be so close to native human CMs that likely only a small-scale parameter fine-tuning is needed for the Land et al. (2017) contractile element integration.

We acknowledge that the calcium handling in the Paci et al. (2020) model inherits parameterisation from the 2018 version, which is based on the dataset by Rast et al. (2015) reporting lower-than-typical cytosolic calcium concentrations. While more recent studies such as Kernik et al. (2019) and Seibertz et al. (2023) indicate higher, more physiologically consistent diastolic and systolic calcium levels in hiPSC-CMs, we would like to retain the original calcium handling formulation for the following reasons:

1. Consistency with the Paci model framework: The electrophysiology component is based on the widely used and validated Paci et al. (2020) model, ensuring compatibility with prior work and comparability with other studies in the field. In addition, Paci model, despite its limitations provides a good framework for drug studies, compared to Kernik (2019) and Koivumäki (2018) models, as discussed by Paci et al. (2021).
2. The Land et al. (2017) mechanical model was re-calibrated against hiPSC-CM contractile output (e.g. contraction amplitude and timing) *in the context of*

the existing calcium transient for this study. Although the calcium handling in the Paci model may differ from the calcium transients observed experimentally in some hiPSC-CM cells, the re-calibration and fitting of the Land model to the calcium transients generated by Paci model allows to reach physiological hiPSC-CM contractility. Therefore, contractility is consistent with calcium dynamics used, even if absolute concentrations may differ from newer experimental estimates.

3. We agree that incorporating updated calcium handling could improve the model, and therefore, we have included this point into the Limitations of the current work (lines 807-816, page 36). For now, such reparameterisation would require retuning both electrophysiological and mechanical components, which is outside the scope of this study. The high variability in hiPSC-CM recordings would also be a challenge for such study. This could all be addressed as future work.

3) About the figures. Many of the figures are either as a whole very difficult to read or contain subpanels that are close to impossible to read. Understandably, there might be some resolution issues, when submitting the figures. However, that does not appear to be the main issue with the figures of this manuscript. Some specific notes:

   * Of the figures that include plotted traces (Figures 2, 5-8) only Figure 6 has panel sizes and text font sizes that support the readability of the figure.

   * Please, try to make good use of the available space in the figure layouts to illustrate the data in as informative way as possible, instead of having so much white space between the panels. The worst example of not doing that is Figure 2, where the x- and y-axes weirdly truncated. The axes could be stretched at least 2-fold or maybe even 2.5-fold, to use the white space in more meaningful way.

   * What is the point of repeating the same legend multiple times in one figure, if the legend is the same for all panels? This applies to Figure 6 and 7.

   * Figure 2: Try to condense the legend texts, so that they do not take so much space in the figure. Figure 2D-F: Please add biomarker values for the calibrated model for CTTP, CTD50, APD50, and APD90 in a same way as in panels A and B. Figure 2E: Please add experimental range for APD50, to be coherent with panel F.

As per reviewer’s comments, figures 2, 5, 7 (used to be figure 6), 8 (used to be figure 7), and 9 (used to be figure 8) have been updated in the manuscript. Figure 6 has been moved from the Supplementary Materials to the main text.

4) Please state clearly the compliance with the human and/or animal ethics

policy of the Journal and provide the required information. Now, there is only a brief note on lines 266-267 that "...human induced pluripotent stem cells (hiPSCs; M398 line (19)) were cultured as described before(20, 21)." To my understanding that does not meet requirements of the Journal.

This has been updated in the manuscript (page 22, line 438-442) clarifying that the experiments in the lab were conducted on the hiPSC cell lines (and no reprogramming from human cells took place in the lab) and indicating that the experiments comply with the ethics policies for working with cell lines. The methods describing hiPSC cell handling and differentiation process have been extended and updated (page 22-23).

Referee #2.

1) The derivation of TRPN and CMDN concentrations assumes proportionality from the adult ToR-ORd model (Tomek et al., 2017), despite known differences in calcium handling between hiPSC-CMs (fetal-like) and adult cardiomyocytes. For example, hiPSC-CMs exhibit reduced SERCA2a activity and higher reliance on transsarcolemmal calcium flux compared to adult cells.

We acknowledge that hiPSC-CMs exhibit immature calcium handling compared to adult cardiomyocytes. The scaling of troponin (TRPN) and calmodulin (CMDN) buffering concentrations from the adult ToR-ORd model was performed as an approximation in the absence of quantitative, hiPSC-CM-specific data. We have now added this point to the Limitations section (page 45, lines 927-929).

2) The calibration uses pooled experimental data from heterogeneous sources (Mixed 2D monolayers and 3D engineered heart tissues), variable pacing rates (0.2-2 Hz) and different temperatures (RT vs. 37{degree sign}C). How did the authors account for inter-experimental variability. Would this approach risk overfitting to non-generalizable conditions?

We interpret the concern as referring to potential implicit bias introduced by fitting to mixed-condition data. While the heterogeneity reduces the risk of overfitting in the classical sense, which typically arises when a model is optimised to narrowly constrained data, it may limit the ability of the model to generalise to any one experimental setup. This is an inherent trade-off in the absence of standardised data and is acknowledged as a limitation. While we did not explicitly account for inter-experimental variability, we mitigated risks of bias by using distinct datasets for calibration and validation. Nonetheless, this has been included in the Limitations section now (page 36, lines 809-814).

3) How were the weights assigned for the weighted sum for TP, RT50, etc.?

The weights have been assigned based on the original equation used for calibration by Land et al. (2017). This has now been clarified in the manuscript (page 13-14).

4) I am quite confused by the MaxAT values during pacing. Experimental values are an order of magnitude different.

We agree that the simulated MaxAT values differ from some experimental reports. This discrepancy likely reflects variability across published hiPSC-CM datasets, as some report MaxAT around 0.2 kPa, and some around 6.5 kPa, with simulated values being 0.4 kPa. This variability in experimental data likely stems from the differences in cell maturity state, differentiation protocol, culture conditions, and measurement techniques.

5) I can't find details on the doses used in the simulation and/or experimental available data used for comparison

This has been updated in the text providing additional details for the data used for comparison (page 12-13, page 20).

6) It would be nice to see experimental validation also of 1 or 2 drugs that are in agreement and 1 or 2 that are not in agreement to clarify whether your experimental validation pipeline is comparable to the literature. What was the temperature used for the simulations of the 63 drugs? Were the experimental data (from the literature) generated at the same temperature?

The simulations of the 63 drugs were performed at 37 °C, and this has now been clarified in the manuscript (page 21). The experimental and clinical data used for comparison were obtained under varying conditions (e.g. temperature, cell type, and measurement protocols), and these have now been specified in the text. We agree that including additional experimental validation would strengthen the study. This is acknowledged as a limitation and identified as an area for future work (page 36, lines 824-828).

7) The validating experiments (and simulations) at 20C are bit underwhelming. Also, it is a very good practice to validate experiments on multiple hiPSC lines.

We agree that validating across multiple hiPSC-CM lines and physiological temperature would strengthen the robustness of the model, and we have included this as future work in the Limitations (page 36, lines 821-824). We make the model open source so other groups can evaluate and alter the model based on their own experimental conditions and cell lines.

8) In the methods: "Differentiation into cardiomyocytes was induced in a monolayer as described previously." no citation is provided. Also how old were the cells at the moment of experiments? Where the cells tested for purity? Any maturation strategy used?

The citation now has been included, and the text has been updated to include the day post-differentiation the cells were used. The purity of the cells was not measured but only the cells that were spontaneously beating were selected for the experiments. No maturation medium or mechanical loading strategies were used (page 22-23).

Dear Dr Folkmanaite,

Re: JP-RP-2025-288623R1 "**Electromechanical Modelling and Simulation of hiPSC-Derived Cardiomyocytes Predict Drug Induced Contractility Effects**" by Milda Folkmanaite, Xin Zhou, Andreas Koschinski, Wan-Hua Hong, Marilù Casini, Manuel Koch, Rémi Peyronnet, Ursula Ravens, Manuela Zaccolo, and Blanca Rodriguez

Thank you for submitting your manuscript to The Journal of Physiology. It has been assessed by a Reviewing Editor and by 2 expert referees and we are pleased to tell you that it is potentially acceptable for publication following satisfactory major revision.

Please advise your co-authors of this decision as soon as possible.

The referee reports are copied at the end of this email.

Please address all the points raised and incorporate all requested revisions or explain in your Response to Referees why a change has not been made. We hope you will find the comments helpful and that you will be able to return your revised manuscript within 2 months. If you require longer than this, please contact journal staff: jp@physoc.org. Please note that this letter does not constitute a guarantee for acceptance of your revised manuscript.

Your revised manuscript should be submitted online using the link in your Author Tasks: Link Not Available. This link is accessible via your account as Corresponding Author; it is not available to your co-authors. If this presents a problem, please contact journal staff (jp@physoc.org). Image files from the previous version are retained on the system. Please ensure you replace or remove any files that are being revised.

If you do not wish to submit a revised version of your manuscript, you must inform our journal staff (jp@physoc.org) or reply to this email to request withdrawal. Please note that a manuscript must be formally withdrawn from the peer review process at one journal before it may be submitted to another journal.

TRANSPARENT PEER REVIEW POLICY: To improve the transparency of its peer review process, The Journal of Physiology publishes online as supporting information the peer review history of all articles accepted for publication. Readers will have access to decision letters, including Editors' comments and referee reports, for each version of the manuscript, as well as any author responses to peer review comments. Referees can decide whether or not they wish to be named on the peer review history document.

ABSTRACT FIGURES: Authors are expected to use The Journal's premium BioRender account to create/redraw their Abstract Figures. Information on how to access this account is here: https://physoc.onlinelibrary.wiley.com/journal/14697793/biorender-access.

This will enable Authors to create and download high-resolution figures. If authors have used the free BioRender service, they can use the instructions provided in the link above to download a high-resolution version suitable for publication.

The link provided should only be used for the purposes of this submission. Authors will be charged for figures created on this account if they are not related to this manuscript submission.

LANGUAGE EDITING AND SUPPORT FOR PUBLICATION: If you would like help with English language editing, or other article preparation support, Wiley Editing Services offers expert help, including English Language Editing, as well as translation, manuscript formatting, and figure formatting at www.wileyauthors.com/eeo/preparation. You can also find resources for Preparing Your Article for general guidance about writing and preparing your manuscript at www.wileyauthors.com/eeo/prepresources.

RIGOUR AND REPRODUCIBILITY: We recommend authors consult The Journal's Rigour and Reproducibility requirements to ensure that their manuscript is suitable for publication: https://physoc.onlinelibrary.wiley.com/pb-assets/hub-assets/physoc/documents/TJP-Rigour-and-Reproducibility-Requirements.pdf

REVISION CHECKLIST:

Check that your Methods section conforms to journal policy: https://jp.msubmit.net/cgi-bin/main.plex?form_type=display_requirements#methods.

Check that data presented conforms to the statistics policy: https://jp.msubmit.net/cgi-bin/main.plex?form_type=display_requirements#statistics.

Upload a full Response to Referees file. To create your 'Response to Referees': copy all the reports, including any comments from the Senior and Reviewing Editors, into a Microsoft Word, or similar, file and respond to each point, using font or background colour to distinguish comments and responses and upload as the required file type.

Please upload two versions of your manuscript text: one with all relevant changes highlighted and one clean version with no changes tracked. The manuscript file should include all tables and figure legends, but each figure/graph should be uploaded as separate, high-resolution files.

You may also upload:

- 'Potential Cover Art' for consideration as the issue's cover image
- Appropriate Supporting Information (Video, audio or data set: see https://jp.msubmit.net/cgi-bin/main.plex?form_type=display_requirements#supp).

We look forward to receiving your revised submission.

If you have any queries, please reply to this email and we will be pleased to advise.

Yours sincerely,

Bjorn Knollmann
Senior Editor
The Journal of Physiology

EDITOR COMMENTS

Reviewing Editor:

While mainly satisfactory, there are a couple of areas in which the revision fell short of meeting the reviewers' expectations:

1. Reviewer #1 is not convinced by the authors' rationale for maintaining a model formulation that predicts non physiological Ca2+ concentration. The reviewer offers useful suggestions to begin to address this issue.

2. Reviewer #2 wants to see a clear link to the literature drug by drug.

Both reviewers maintain that the manuscript may represent an important contribution to the field after the concerns are appropriately addressed. The reviewing editor concurs.

Senior Editor:

I concur with the reviewing editor's assessment.

REFEREE COMMENTS

Referee #1:

Folkmanaite et al. have properly addressed three of the four major concerns that I raised in my 1st round review. Please, see the point-by-point details below (not in numerical order).

Authors' response to concern #1 (about the novelty of the model):

"The wording has been edited as per the referee's suggestion. The text (lines 756 - 782, pages 34-35) has been expanded to include the differences between Rice and Land models."

This is OK on my behalf, no further comments on this.

Authors' response to concern #3 (about the figures):

"As per reviewer's comments, figures 2, 5, 7 (used to be figure 6), 8 (used to be figure 7), and 9 (used to be figure 8) have been updated in the manuscript. Figure 6 has been moved from the Supplementary Materials to the main text."

In Figure 2, the overly long legend texts still seem to take about one fourth of the figure width. However, the updated version is much more readable than the original. So, I have no further comments on this.

Authors' response to concern #4 (about the ethics):

"This has been updated in the manuscript (page 22, line 438-442) clarifying that the experiments in the lab were conducted on the hiPSC cell lines (and no reprogramming from human cells took place in the lab) and indicating that the experiments comply with the ethics policies for working with cell lines. The methods describing hiPSC cell handling and differentiation process have been extended and updated (page 22-23)."

The extended description is OK. No further comments on this.

Authors' response to concern #2 (about the credibility of the model):

"We acknowledge that the calcium handling in the Paci et al. (2020) model inherits parameterisation from the 2018 version, which is based on the dataset by Rast et al. (2015) reporting lower-than-typical cytosolic calcium concentrations. While more recent studies such as Kernik et al. (2019) and Seibertz et al. (2023) indicate higher, more physiologically consistent diastolic and systolic calcium levels in hiPSC-CMs, we would like to retain the original calcium handling formulation for the following reasons:

1. Consistency with the Paci model framework: The electrophysiology component is based on the widely used and validated Paci et al. (2020) model, ensuring compatibility with prior work and comparability with other studies in the field.

2. The Land et al. (2017) mechanical model was re-calibrated against hiPSC-CM contractile output (e.g. contraction amplitude and timing) in the context of the existing calcium transient for this study. Although the calcium handling in the Paci model may differ from the calcium transients observed experimentally in some hiPSC-CM cells, the re-calibration and fitting of the Land model to the calcium transients generated by Paci model allows to reach physiological hiPSC-CM contractility. Therefore, contractility is consistent with calcium dynamics used, even if absolute concentrations may differ from newer experimental estimates.

3. We agree that incorporating updated calcium handling could improve the model, and therefore, we have included this point into the Limitations of the current work (lines 807-816, page 36). For now, such reparameterisation would require retuning both electrophysiological and mechanical components, which is outside the scope of this study. The high variability in hiPSC-CM recordings would also be a challenge for such study. This could all be addressed as future work."

Reason #1 goes strongly against the basic philosophy of science as an iterative and self-correcting process. In essence, the authors are stating that it is more important to ensure sort of an "engineering compatibility" with the Paci et al. (2020) model and related body of work, instead of correcting a known physiological inconsistency in the model. In my understanding, the Journal of Physiology emphasises correct representation of physiology in modelling.

Reason #2: This not a proper justification. What is the value of a correctly-scaled output, when the input is incorrect? Or in other words, what is the physiological relevance of re-calibrating and integrating the Land et al. CE model, when the intracellular Ca2+ linking the electrophysiological and mechanical components is known to be wrong?

Reason #3 is contradicting. Opting to use the unphysiological calcium dynamics is not in line with, what the authors write in their Conclusions (lines 833-835 in the manuscript): "We describe the development, calibration and validation of a new hiPS-CM electromechanical model for the simultaneous simulation of drug effects on active tension, calcium transient and cellular electrophysiology."

To conclude, please, reparameterise the intracellular calcium handling of the Paci et al. (2020) model, so that the simulated [Ca2+] amplitude and dynamics match the current experimental body of evidence. Specifically, employ the previously mentioned Kernik et al. (2019) and Seibertz et al. (2023) in vitro data, and other possibly available coherent data sets. I do realise that the reparameterisation requires effort. However, it is a worthy and timely update to the "Paci lineage" of hiPSC-CM models, because intracellular Ca2+ concentration is such a central variable in excitation-contraction coupling. As you know, the absolute concentration values are relevant for not only contractility but also, for example, calcium-dependent inactivation of L-type Ca2+ channel, activation of Na+/Ca2+ exchanger, etc.

One potentially helpful small note for you consideration: When you reparameterise the intracellular calcium handling of the Paci et al. (2020) model, the resulting increase of calcium-dependent inactivation of L-type Ca2+ channel (going from basically non-existing to physiological regime) might cause problems in AP plateau phase. However, that is not a problem of calcium dynamics nor the parameters of I_CaL. At least that is what we concluded, when we were updating the Koivumäki et al. (2018) hiPSC-CM model in our recent study (Naumenko et al. 2024; https://doi.org/10.1016/j.yjmcc.2024.06.003). Instead, in our understanding, the problem was the I_K1 formulation. The Ma et al. (2011) data set, which Paci et al. (2015) originally used to parameterise I_K1, has likely some other contaminating potassium current, because the I-V curve does not look like it should for I_K1. Unfortunately, these kinds of technical errors combined with lack of understanding of cardiomyocyte electrophysiology appears to be quite common in the early in vitro hiPSC-CM data sets. In the Naumenko et al. (2024) study, we opted to use the I_K1 formulation by Carro et al. (2011), which has been used also in some of the recent hv-CM modelling work from your lab. You can check out the details of our approach in the supplementary material of the Naumenko et al. (2024) paper, if you find them helpful.

Referee #2:

The authors have responded to all my concerns. Most of my requests have been added in the limitation section and no more results have been added to address some of my points. I appreciate the extensive table on the literature used, but I am still missing a clear link between the drug and the literature. For example, if I look at Fig 4, Diazepam is in disagreement between experimental and in silico modeling. What is the exact citation used by the authors for diazepam? And Amiodarone? Or any other drug? Could the authors clarify where is the experimental data for each molecule coming from? Considering that the authors are not further validating their pipeline using drugs with known effects, a clear link to the literature drug by drug would be important. If it's there and I missed it, I apologize.

END OF COMMENTS

EDITOR COMMENTS

Reviewing Editor:

While mainly satisfactory, there are a couple of areas in which the revision fell short of meeting the reviewers' expectations:

1. Reviewer #1 is not convinced by the authors' rationale for maintaining a model formulation that predicts non physiological Ca2+ concentration. The reviewer offers useful suggestions to begin to address this issue.

2. Reviewer #2 wants to see a clear link to the literature drug by drug.

Both reviewers maintain that the manuscript may represent an important contribution to the field after the concerns are appropriately addressed. The reviewing editor concurs.

Senior Editor:

I concur with the reviewing editor's assessment.

REFEREE COMMENTS

Referee #1:

Folkmanaite et al. have properly addressed three of the four major concerns that I raised in my 1st round review. Please, see the point-by-point details below (not in numerical order).

Authors' response to concern #1 (about the novelty of the model):

"The wording has been edited as per the referee's suggestion. The text (lines 756 - 782, pages 34-35) has been expanded to include the differences between Rice and Land models."

This is OK on my behalf, no further comments on this.

Authors' response to concern #3 (about the figures):

"As per reviewer's comments, figures 2, 5, 7 (used to be figure 6), 8 (used to be figure 7), and 9 (used to be figure 8) have been updated in the manuscript. Figure 6 has been moved from the Supplementary Materials to the main text."

In Figure 2, the overly long legend texts still seem to take about one fourth of the figure width. However, the updated version is much more readable than the original. So, I have no further comments on this.

Authors' response to concern #4 (about the ethics):

"This has been updated in the manuscript (page 22, line 438-442) clarifying that the experiments in the lab were conducted on the hiPSC cell lines (and no reprogramming from human cells took place in the lab) and indicating that the experiments comply with the ethics policies for working with cell lines. The methods describing hiPSC cell handling and differentiation process have been extended and updated (page 22-23)."

The extended description is OK. No further comments on this.

Authors' response to concern #2 (about the credibility of the model):

"We acknowledge that the calcium handling in the Paci et al. (2020) model inherits parameterisation from the 2018 version, which is based on the dataset by Rast et al. (2015) reporting lower-than-typical cytosolic calcium concentrations. While more recent studies such as Kernik et al. (2019) and Seibertz et al. (2023) indicate higher, more physiologically consistent diastolic and systolic calcium levels in hiPSC-CMs, we would like to retain the original calcium handling formulation for the following reasons:

1. Consistency with the Paci model framework: The electrophysiology component is based on the widely used and validated Paci et al. (2020) model, ensuring compatibility with prior work and comparability with other studies in the field.

2. The Land et al. (2017) mechanical model was re-calibrated against hiPSC-CM contractile output (e.g. contraction amplitude and timing) in the context of the existing calcium transient for this study. Although the calcium handling in the Paci model may differ from the calcium transients observed experimentally in some hiPSC-CM cells, the re-calibration and fitting of the Land model to the calcium transients generated by Paci model allows to reach physiological hiPSC-CM contractility. Therefore, contractility is consistent with calcium dynamics used, even if absolute concentrations may differ from newer experimental estimates.

3. We agree that incorporating updated calcium handling could improve the model, and therefore, we have included this point into the Limitations of the current work (lines 807-816, page 36). For now, such reparameterisation would require retuning both electrophysiological and mechanical components, which is outside the scope of this study. The high variability in hiPSC-CM recordings would also be a challenge for such study. This could all be addressed as future work."

Reason #1 goes strongly against the basic philosophy of science as an iterative and self-correcting

process. In essence, the authors are stating that it is more important to ensure sort of an "engineering compatibility" with the Paci et al. (2020) model and related body of work, instead of correcting a known physiological inconsistency in the model. In my understanding, the Journal of Physiology emphasises correct representation of physiology in modelling.

Reason #2: This not a proper justification. What is the value of a correctly-scaled output, when the input is incorrect? Or in other words, what is the physiological relevance of re-calibrating and integrating the Land et al. CE model, when the intracellular Ca2+ linking the electrophysiological and mechanical components is known to be wrong?

Reason #3 is contradicting. Opting to use the unphysiological calcium dynamics is not in line with, what the authors write in their Conclusions (lines 833-835 in the manuscript): "We describe the development, calibration and validation of a new hiPS-CM electromechanical model for the simultaneous simulation of drug effects on active tension, calcium transient and cellular electrophysiology."

To conclude, please, reparameterise the intracellular calcium handling of the Paci et al. (2020) model, so that the simulated [Ca2+] amplitude and dynamics match the current experimental body of evidence. Specifically, employ the previously mentioned Kernik et al. (2019) and Seibertz et al. (2023) in vitro data, and other possibly available coherent data sets. I do realise that the reparameterisation requires effort. However, it is a worthy and timely update to the "Paci lineage" of hiPSC-CM models, because intracellular Ca2+ concentration is such a central variable in excitation-contraction coupling. As you know, the absolute concentration values are relevant for not only contractility but also, for example, calcium-dependent inactivation of L-type Ca2+ channel, activation of Na+/Ca2+ exchanger, etc.

One potentially helpful small note for you consideration: When you reparameterise the intracellular calcium handling of the Paci et al. (2020) model, the resulting increase of calcium-dependent inactivation of L-type Ca2+ channel (going from basically non-existing to physiological regime) might cause problems in AP plateau phase. However, that is not a problem of calcium dynamics nor the parameters of I_CaL. At least that is what we concluded, when we were updating the Koivumäki et al. (2018) hiPSC-CM model in our recent study (Naumenko et al. 2024; https://doi.org/10.1016/j.yjmcc.2024.06.003). Instead, in our understanding, the problem was the I_K1 formulation. The Ma et al. (2011) data set, which Paci et al. (2015) originally used to parameterise I_K1, has likely some other contaminating potassium current, because the I-V curve does not look like it should for I_K1. Unfortunately, these kinds of technical errors combined with lack of understanding of cardiomyocyte electrophysiology appears to be quite common in the early in vitro hiPSC-CM data sets. In the Naumenko et al. (2024) study, we opted to use the I_K1 formulation by Carro et al. (2011), which has been used also in some of the recent hv-CM modelling work from your lab. You can

check out the details of our approach in the supplementary material of the Naumenko et al. (2024) paper, if you find them helpful.

Many thanks for the constructive suggestions from the reviewer to improve this paper. In response to the referee's request, we have reparameterised intracellular calcium handling based on recent experimental datasets (Seibertz et al. 2023) and developed a new version of the model (Version 2) that reproduces physiologically realistic calcium amplitudes and dynamics. To reflect experimentally observed heterogeneity in hiPSC-CM maturity, we now present both the original formulation (Version 1, less mature phenotype) and the updated formulation (Version 2, more mature phenotype). All analyses, figures, and conclusions have been updated accordingly.

The new version of the model considered the increased INa, ICaL and IK1 conductances as reported in the Seibertz et al. 2023 study to represent the calcium handling changes in hiPSC-CM over long-term culture with more maturity. With this new version of the model, we reproduced the hiPSC-CM calcium amplitude in range with Garg et al. 2018 and Kernik et al. 2019, along with an increased active tension magnitude closer to adult cardiomyocytes.

Because published experimental datasets report both adult-like calcium amplitudes (e.g. Garg et al. 2018) and markedly lower force generation in many hiPSC-CM preparations (Table 2), we interpret these as representing different maturation states of the hiPSC-CM heterogeneity. We therefore include both model variants to explicitly capture this experimentally observed spectrum. Both versions are now included in the manuscript, with changes reflected in Methods, Figures, and Discussion.

This new version of the model (Version 2) reproduced the same effects for the 7 reference compounds in group 1, consistent with experimental data. With the increase of ICaL conductance, the new version of the model is more sensitive to calcium blockage effects, which favours the prediction towards negative inotropic effects. The simulated accuracy of inotropic effects for the group 2 list of 64 drugs was slightly improved in the new model, reproducing more agreements between simulation and experimental data. Similar the Version 1, rate-dependent difference of paliperidone effects was also observed with the version 2 of the model, highlighting the robustness of the proposed mechanisms.

Referee #2:

The authors have responded to all my concerns. Most of my requests have been added in the limitation section and no more results have been added to address some of my points. I appreciate the extensive table on the literature used, but I am still missing a clear link between the drug and the literature. For example, if I look at Fig 4, Diazepam is in disagreement

between experimental and in silico modeling. What is the exact citation used by the authors for diazepam? And Amiodarone? Or any other drug? Could the authors clarify where is the experimental data for each molecule coming from? Considering that the authors are not further validating their pipeline using drugs with known effects, a clear link to the literature drug by drug would be important. If it's there and I missed it, I apologize.

Thank you for raising this point. The drug-by-drug table was provided in the previous submission as a supporting document; however, we appreciate that this may not have been accessible during review. To ensure clarity and transparency, we have now moved this table into the main manuscript as Table 4. This table lists each compound alongside its corresponding experimental references, for example, Table 4 now explicitly lists the experimental sources used for diazepam and amiodarone.

Dear Dr Folkmanaite,

Re: JP-RP-2026-288623R2 "**Electromechanical Modelling and Simulation of hiPSC-Derived Cardiomyocytes Predict Drug Induced Contractility Effects**" by Milda Folkmanaite, Xin Zhou, Andreas Koschinski, Wan-Hua Hong, Marilù Casini, Manuel Koch, Rémi Peyronnet, Ursula Ravens, Manuela Zaccolo, and Blanca Rodriguez

We are pleased to tell you that your paper has been accepted for publication in The Journal of Physiology.

Please see comments below for some amendments to consider at proof stage.

The last Word (or similar) version of the manuscript provided will be used by the Production Editor to prepare your proof. When this is ready you will receive an email containing a link to Wiley's Online Proofing System. The proof should be thoroughly checked and corrected as promptly as possible.

Authors should note that it is too late at this point to offer corrections prior to proofreading. Major corrections at proof stage, such as changes to figures, will be referred to the Editors for approval before they can be incorporated. Only minor changes, such as to style and consistency, should be made at proof stage. Changes that need to be made after proof stage will usually require a formal correction notice.

All queries at proof stage should be sent to: TJP@wiley.com.

Following copyediting and typesetting the accepted version will be published online.

Yours sincerely,

Bjorn Knollmann
Senior Editor
The Journal of Physiology

IMPORTANT POINTS TO NOTE FOLLOWING ACCEPTANCE OF YOUR PAPER:

• IMPORTANT NOTICE ABOUT OPEN ACCESS: To assist authors whose funding agencies mandate immediate public access to published research findings, The Journal of Physiology allows authors to pay an Open Access (OA) fee to have their papers made freely available immediately on publication.

The Corresponding Author will receive an email from Wiley with details on how to register or log-in to Wiley Authors Services where you will be able to place an order.

• You can check if your funder or institution has a Wiley Open Access Account here: https://authorservices.wiley.com/author-resources/Journal-Authors/licensing-and-open-access/open-access/author-compliance-tool.html.

• TRANSPARENT PEER REVIEW POLICY: To improve the transparency of its peer review process, The Journal of Physiology publishes online as supporting information the peer review history of all articles accepted for publication. Readers will have access to decision letters, including Editors' comments and referee reports, for each version of the manuscript as well as any author responses to peer review comments. Referees can decide whether or not they wish to be named on the peer review history document.

• You can help your research get the attention it deserves! Check out Wiley's free Promotion Guide for best-practice recommendations for promoting your work at: www.wileyauthors.com/eeo/guide. You can learn more about Wiley Editing Services which offers professional video, design, and writing services to create shareable video abstracts, infographics, conference posters, lay summaries, and research news stories for your research at: www.wileyauthors.com/eeo/promotion.

• If you would like to receive our 'Research Roundup', a monthly newsletter highlighting the cutting-edge research published in The Physiological Society's family of journals (The Journal of Physiology, Experimental Physiology, Physiological Reports, The Journal of Nutritional Physiology and The Journal of Precision Medicine: Health and Disease), please click this link, fill in your name and email address and select 'Research Roundup':
https://www.physoc.org/journals-and-media/membernews

EDITOR COMMENTS

Reviewing Editor:

Thank you for a responsive and thorough revision. Congratulations!

Senior Editor:

The manuscript is now acceptable. The authors should consider shortening the legend of table 4 at the proof stage. Thank you for your excellent contribution!

REFEREE COMMENTS

Referee #1:

Folkmanaite et al. have properly addressed the last major concern that remained after the 2nd review round. It would appear that developing the updated model variant ("Version 2", as the authors) with more physiological calcium dynamics was a worthwhile effort, since simulations with "Version 2" identified better the inotropic activity of tested drugs than "Version 1". I appreciate the authors taking the development of "Version 2" as a chance to explore the variety, which, as we know, is high in hiPSC-CMs.

Personally, I would probably have included only the results obtained with "Version 2" of the model to main manuscript, and the "Version 1" results could have been presented in the supplement. However, I can appreciate the authors' preference to display the spectrum, so to speak, by including results from both versions in the main manus.

Also, I would argue that referring to "Version 1" and "Version 2" as less and more mature, respectively, is potentially misleading. It suggests that the lower force generation in many hiPSC-CM preparations would be a result of (and would indicate a) lower intracellular calcium transient (CT) amplitude. That could be characterised as sort of an engineering point of view, where a weaker input (smaller CT amplitude) results in weaker output (less force) in less mature CMs. However, from a biophysical perspective, it seems more likely that the lower force generation in hiPSC-CMs is due to immaturity of the contractile machinery (myofibrillar disarray, lower expression of the proteins, etc.). As the proteins of that machinery are also the major calcium buffer in CMs, one might even expect the CT amplitudes to be higher in hiPSC-CMs than in mature native CMs? But, I acknowledge that expanding these considerations are beyond the scope of this manus.

I have no further comments.

Referee #2:

The authors have responded to my points. The legend of table 4 is incredibly long. Is it really necessary to list the citations like that?
